# Research progress of targeted therapy regulating Th17/Treg balance in bone immune diseases

**DOI:** 10.3389/fimmu.2024.1333993

**Published:** 2024-01-30

**Authors:** Xiaxia Wang, Bai Sun, Yujie Wang, Peng Gao, Jiayi Song, Weirong Chang, Zhipan Xiao, Yongbin Xi, Zhonghong Li, Fangyu An, Chunlu Yan

**Affiliations:** ^1^ School of Traditional Chinese and Western Medicine, Gansu University of Chinese Medicine, Lanzhou, Gansu, China; ^2^ School of Basic Medicine, Gansu University of Chinese Medicine, Lanzhou, Gansu, China; ^3^ Orthopaedics Department, The No.2 People’s Hospital of Lanzhou, Lanzhou, Gansu, China; ^4^ Pathological Research Centre, Gansu University of Chinese Medicine, Lanzhou, Gansu, China; ^5^ Teaching Experiment Training Centre, Gansu University of Chinese Medicine, Lanzhou, Gansu, China

**Keywords:** rheumatoid arthritis, postmenopausal osteoporosis, targeted therapy, Th17/Treg equilibrium, bone immunity

## Abstract

Rheumatoid arthritis (RA) and postmenopausal osteoporosis (PMOP) are common bone-immune diseases. The imbalance between helper (Th17) and regulatory T cells (Tregs) produced during differentiation of CD4^+^ T cells plays a key regulatory role in bone remodelling disorders in RA and PMOP. However, the specific regulatory mechanism of this imbalance in bone remodelling in RA and PMOP has not been clarified. Identifying the regulatory mechanism underlying the Th17/Treg imbalance in RA and PMOP during bone remodelling represents a key factor in the research and development of new drugs for bone immune diseases. In this review, the potential roles of Th17, Treg, and Th17/Treg imbalance in regulating bone remodelling in RA and PMOP have been summarised, and the potential mechanisms by which probiotics, traditional Chinese medicine compounds, and monomers maintain bone remodelling by regulating the Th17/Treg balance are expounded. The maintenance of Th17/Treg balance could be considered as an therapeutic alternative for the treatment of RA and PMOP. This study also summarizes the advantages and disadvantages of conventional treatments and the quality of life and rehabilitation of patients with RA and PMOP. The findings presented her will provide a better understanding of the close relationship between bone immunity and bone remodelling in chronic bone diseases and new ideas for future research, prevention, and treatment of bone immune diseases.

## 1 Introduction

Bone is a dynamic organ that maintains its proper structure and function through continuous remodelling throughout the life cycle of an organism (1). The immune system plays an important role in the progression of autoimmune diseases because of its inherent adaptive components (2). Bone and immune cells share common progenitor cells i.e., bone marrow stromal cells, and have many common regulatory factors that not only affect bone cells but also regulate immune lineage cells. Therefore, bone immunology has emerged as a new interdisciplinary subject for studying rheumatoid arthritis (RA) and postmenopausal osteoporosis (PMOP) (3, 4).

**Figure 1:**
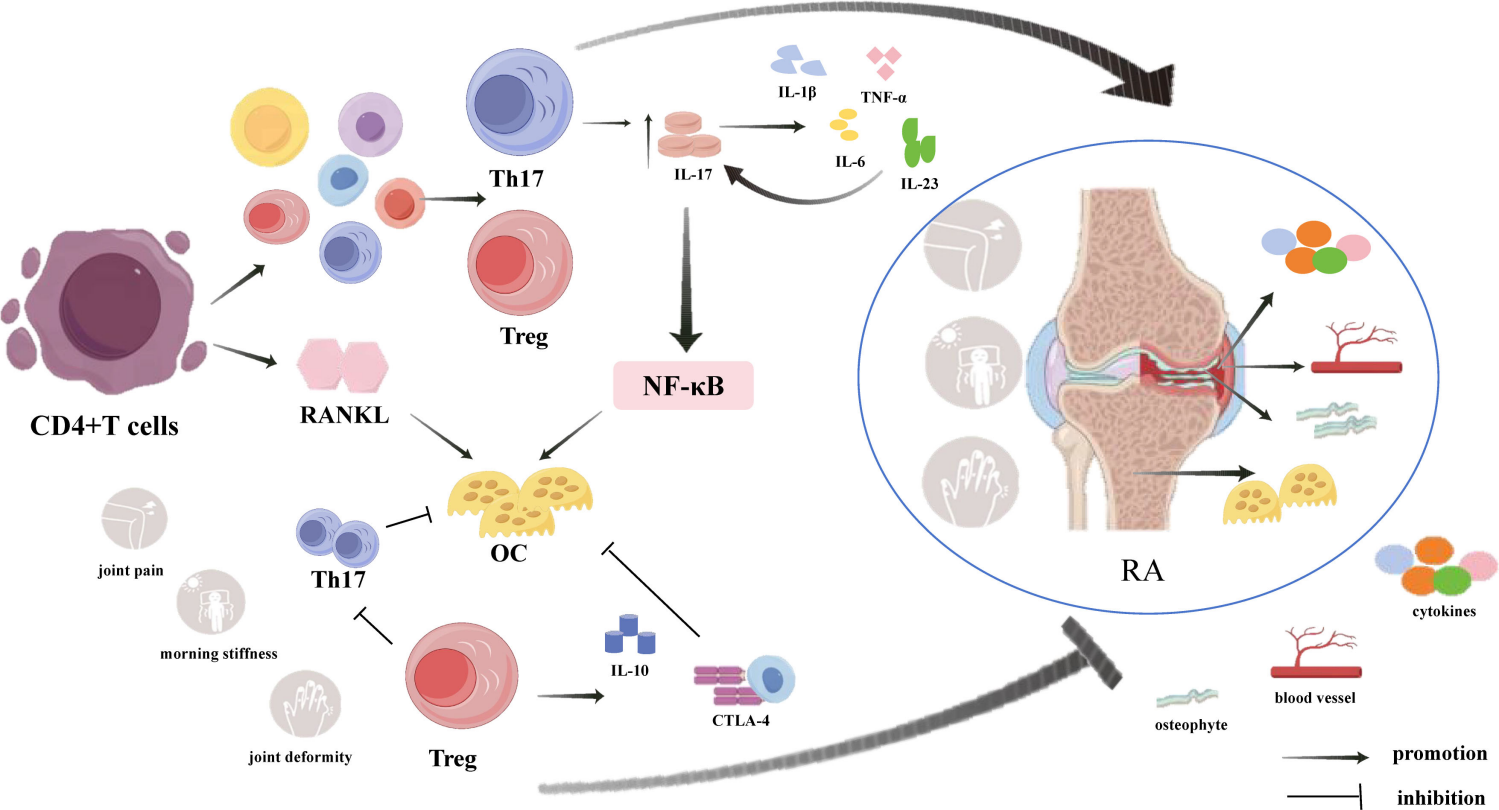
Mechanisms of CD4+T cells regulating inflammatory response and OC generation in RA through secretion of Th17 and Treg cells. In addition to secreting RANKL to directly promote OC differentiation, CD4+T cells also regulate inflammatory responses and OC production mainly by secreting Th17 cells and Treg cells.Th17 cells and their secreted cytokines play pro-inflammatory and pro-OC differentiation roles, while Treg cells and their secreted cytokines play anti-inflammatory roles and inhibit OC and Th17 cell differentiation, suggesting that the balance between Th17 cells and Treg cells influences the development of RA.

RA is a chronic and progressive autoimmune disease characterised by multiple symmetrical joint leukocyte infiltration and systemic osteoporosis (5, 6). Pathological changes include synovial hyperplasia, angiogenesis, pannus formation, inflammatory cell infiltration, articular cartilage, and bone destruction, leading to joint dysfunction and deformity (7, 8). Clinically, joint pain, tenderness, and rigidity are often accompanied by immune osteoporosis. Irreversible joint injury gradually appears, with joint movement disorders and deformities occurring at later stages (9). Epidemiological investigations have shown that the total incidence of RA worldwide is 1–2%. When treatment is delayed, the disability rate in patients with RA within 2–3 years can reach 0.5–1% (10–12). Currently, antirheumatic drugs are often used clinically to control inflammation and delay disease progression. However, this routine treatment has many adverse effects and does not produce obvious therapeutic effect in many patients (13). Therefore, novel treatment strategies for RA need to be developed.

the pathogenesis of RA is extremely complex and involves many immune factors, T cell dysfunction, which plays a vital role in the occurrence and development of RA (14). During the immune response, naïve CD4^+^T cells are activated and differentiate into T cell subsets, mainly helper T cells (Th17) and regulatory T cells (Tregs), which are important triggers for local and systemic inflammation and bone loss in RA (15, 16). They can affect the inflammatory process and the activation and differentiation of osteoblasts (OBs) and osteoclasts (OCs) by regulating a variety of cytokines that are closely related to bone remodelling (**Figure 1**) (17). In RA, Th17 cells secrete proinflammatory cytokine IL-17, which can promote the production of TNF-α, IL-1β, IL-6 and IL-23, which in turn promote the secretion of IL-17, thereby aggravates the inflammatory reaction and forms a complex inflammatory network (18–20). OCs is the main cause of bone destruction (21). And in RA, activated T cells subsets, such as Th1, Th17, Th9, and Th22, can express RANKL in a direct or indirect way to stimulate the differentiation and maturation of OC (22). Moreover, Th17 cells could secrete IL-17, which promotes cartilage degradation and destruction, and at the same time further activates OC through the NF-κB pathway, resulting in an imbalance of bone remodelling (23, 24). Compared with Th17 cells, Tregs inhibit the inflammatory response and RANKL-induced OC production through two different cytokine-dependent mechanisms: IL-10 and cell-cell contact through CTLA-4 (25, 26). In addition, Tregs can inhibit excessive immune responses and play an important role in preventing Th17 cells activation (27). The number and functional impairment of Tregs are among the main factors in RA (28). Therefore, the imbalance and dysfunction of Tregs and Th17 cells are related to the pathogenesis of RA.

**Figure 2:**
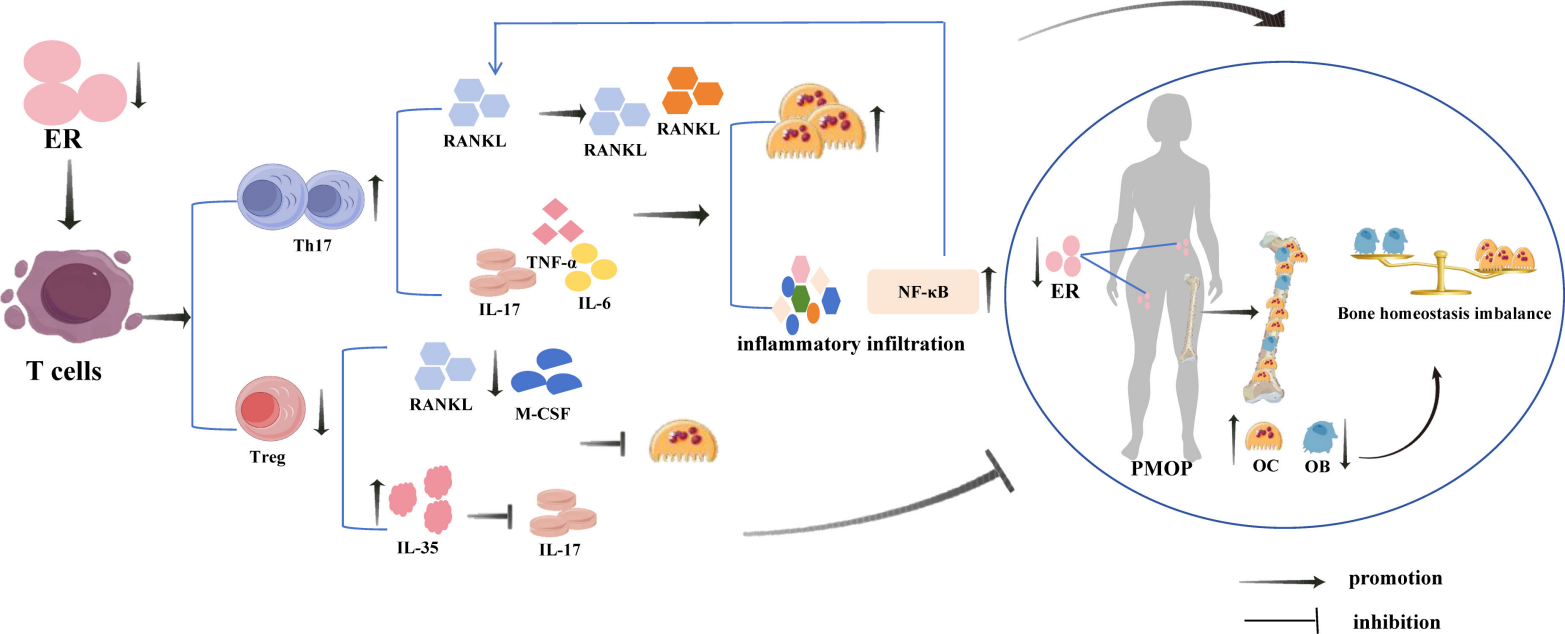
Estrogen deficiency induces T cell differentiation and regulatory effects of Th17 and Treg cells on PMOP. Oestrogen deficiency activates T cells to secrete a large number of Th17 cells and a smaller number of Treg cells, in which Th17 cells and the cytokines they secrete play a pro-inflammatory and pro-OC generation role, and Treg cells and the related cytokines they secrete play an anti-inflammatory and anti-OC generation role. However, the pro-inflammatory and pro-OC generation of Th17 cells far exceeded the anti-inflammatory and anti-bone resorption effects of Treg cells, which made the inflammatory response and bone resorption far greater than bone formation, leading to the development of PMOP.

PMOP is a systemic metabolic bone disease caused by a sudden decrease in oestrogen levels in postmenopausal women and characterised by bone mass reduction and microstructural destruction, resulting in a decrease in bone strength and an increase in bone fragility (23, 29). It is considered a serious public health problem that poses a significant economic burden on society (30). In recent years, the regulation of bone metabolism by the immune system has been of wide concern, and research on the immune system’s role in osteoporosis has led to the creation of the new field “immunoporosis” (31). Activated T cells have been found to participate in bone remodelling together with other immune cytokines under chronic inflammation caused by oestrogen deficiency (**Figure 2**) (32), and overactivated T cells stimulate the formation of OCs and accelerate bone resorption by secreting OC-promoting factors IL-17, IL-6, TNF-α and RANKL (33, 34) In addition, in ovariectomized mice, the Th17 cell number increased significantly, Treg cell number decreased significantly, and the Th17/Treg cell ratio became unbalanced (35). Th17 cells have opposite effects to those of Treg cells; Th17 cells are a pro-inflammatory T cell subset, which not only directly express RANKL and promote the combination of RANKL and RANK, but also stimulate OC to produce Sertoli cells by secreting inflammatory factors, such as IL-17, TNF-α, and IL-6, promoting inflammatory infiltration, and increasing the expression of NF-κB, further up-regulating the expression of RANKL and stimulating the maturation and differentiation of OC (17). In contrast, Treg cells inhibit the expression of RANKL and M-CSF, and also secrete IL-35 and reduce the production of IL-17, thereby directly or indirectly inhibiting OC production through a cytokine-dependent mechanism. Additionally, Tregs bind to OC precursors through direct contact and inhibit OC (36, 37). However, under the pathological conditions of PMOP oestrogen deficiency, Tregs lose their immunosuppressive function and transform into Th17 cells, which further promote OC differentiation, lead to bone resorption exceeding bone formation, and induce a bone remodelling imbalance (38). Therefore, an imbalance in Th17/Treg cells is important for the pathogenesis of PMOP, and regulating this balance is expected to provide a new opportunity to treat PMOP. Currently, oestrogen replacement therapy is the main therapeutic drug for PMOP; however, in addition to its therapeutic effects, it causes a series of carcinogenic risks, thus reducing its acceptance by patients (39). Therefore, safe and effective anti-PMOP agents are needed.

In recent years, with the development of network pharmacology and molecular docking technologies, probiotics and traditional Chinese medicines have been found to have unique advantages in regulating the interactions between the immune system and RA and PMOP (40–42). Probiotics regulate the balance of Th17/Treg cells by regulating the “intestine-immunity-bone axis” and play therapeutic roles in RA and PMOP (40, 43). Traditional Chinese medicine compounds and monomers also provide new ideas and opportunities for developing syndrome differentiation and immunotherapy targets for RA and PMOP by regulating Th17/Treg cells (44, 45). Based on this, this paper summarises the relationship between immune bone remodelling and RA and PMOP and describes the regulation of immune bone homeostasis through drug-targeted regulation of the Treg/Th17 cell balance to provide new ideas for follow-up research and clinical treatment.

## 2 Regulatory factors associated with Th17 and Treg differentiation

Under different conditions, initially, CD4+T cells are activated and differentiate into different T cell (Th) subsets, namely, Th1, Th2, Th17, and Treg cells (46). Among the T cell subsets, Th17 and Treg are the most representative (47), and they are involved in the occurrence and development of several diseases, such as cancer, autoimmune diseases, and metabolic diseases (48, 49). And studies have confirmed that stimulation of Th17 and Treg differentiation is closely related to inflammatory factors, cytokines and transcription factors and signalling pathways.

### 2.1 Regulation of inflammatory factors affecting differentiation of Th17 and Treg

**Figure 3:**
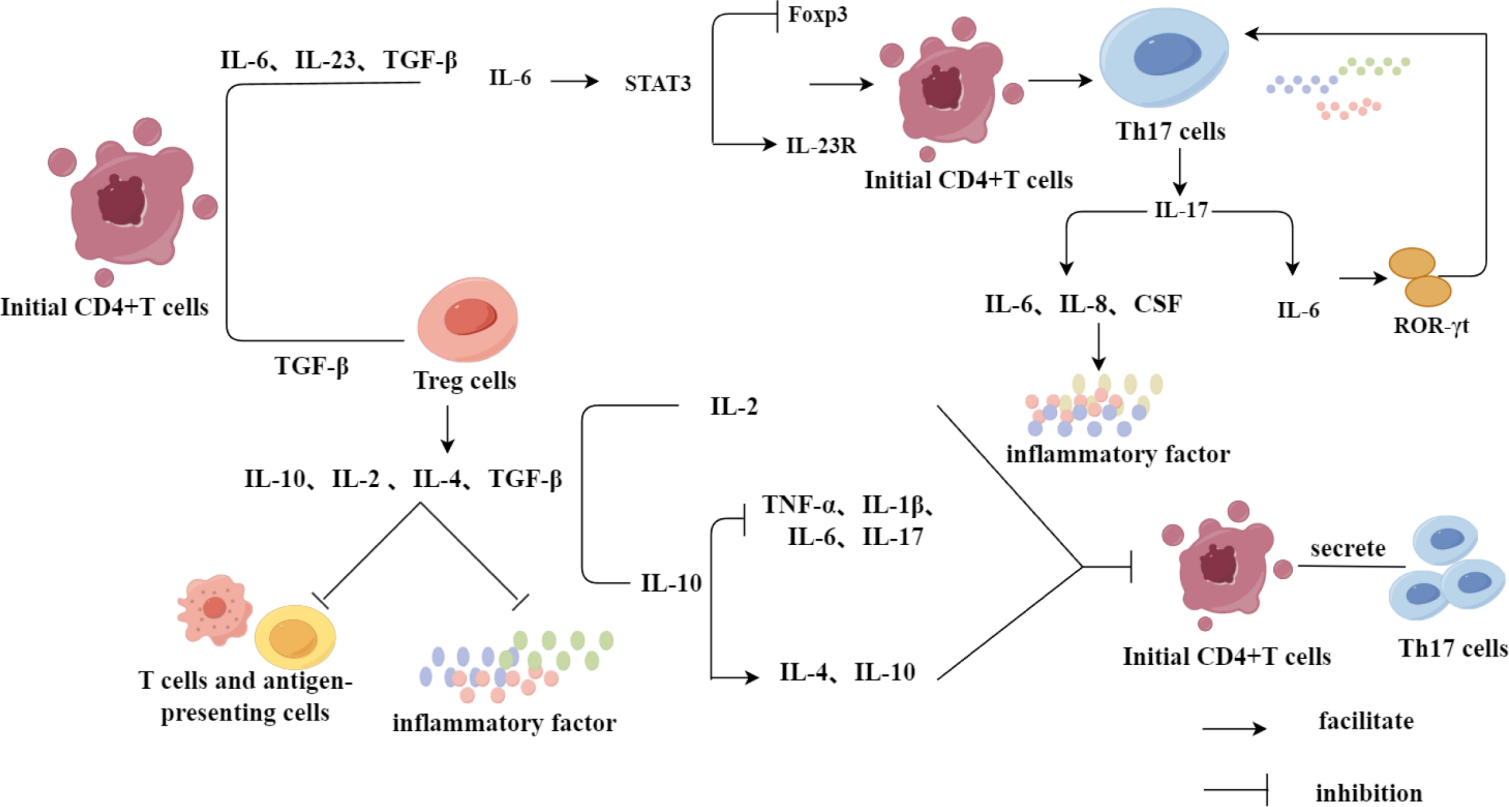
Regulation of Th17 and Treg differentiation by inflammatory factors. Initial CD4+T cells were differentiated into Treg cells under the induction of TGF-β, in which Treg cells were not only able to inhibit the inflammatory response and play an immunosuppressive role through the secretion of relevant inflammation-suppressing factors, but also inhibited the differentiation of initial CD4+T cells to Th17 cells. While the initial CD4+T cells, in the presence of IL-6, IL-23 and TGF-β together, promoted the differentiation of CD4+T cells to Th17 cells through a series of IL-6’s mechanism of action in a direct or indirect way.

Initial CD4+T cells are induced to differentiate into Treg cells via transforming growth factor-β (TGF-β); while immature T cells are induced to differentiate into Th17 cells by the upregulation of IL-23R and the combined action of IL-6, IL-23, and TGF-β, with IL-6 inhibiting the expression of Foxp3 by activating signal transducer and activator of transcription 3 (STAT3) (50–52). Th17 cells produce various inflammatory cytokines, the most important of which is IL-17 (53). As a proinflammatory mediator, IL-17 can further stimulate the expression of IL-6, IL-8, and colony-stimulating factor (CSF), and mediate the infiltration of inflammatory cells and tissue damage (54). In addition, the differentiation of Th17 cells by the cytokines IL-17 and TNF-α is regulated by the expression of its specific transcription factor retinoic acid-related orphan receptor (ROR-γt), while the differentiation of these cells by IL-6 is based on the upregulation of ROR-γt expression and initiation of the ROR-γt signal transduction pathway (55). Further, Treg cells can secrete anti-inflammatory cytokines, such as IL-10, IL-2, IL-4, and TGF-β, inhibit T cells and antigen presenting cells, and play an immunosuppressive role by reducing the secretion of pro-inflammatory cytokines (31). IL-10 blocks the production of TNF-α, IL-1β, IL-6 and IL-17 by inhibiting the response of highly pathogenic Th17 cells, which simultaneously increases the levels of IL-4 and IL-10 (56, 57). IL-2 maintains the survival of Tregs by inhibiting the differentiation of CD4+T cells into Th17 cells (58). TGF-β, as a common regulatory factor for the differentiation of initial CD4+T cells into Th17 and Treg cells, is very important for driving the imbalance in the Th17/Treg cell ratio (**Figure 3**) (50).

Methylene tetrahydrofolate dehydrogenase 2 (MTHFD2), a single carbon (1C)-metabolising enzyme, supports T cell growth and division, while the lack of MTHFD2 has different effects on T cell differentiation and effector function in each subgroup tested, although RORgt expression did not change in Th17 cells, it induces the expression of FoxP3 in Th17 cells and enhanced Treg cell differentiation. MTHFD2 can also promote the differentiation of Treg cells in environments with a low concentration of TGF-β (59). Wu et al. (60) found that SGK1, a key kinase, regulates the balance between Tregs and Th17 cells by activating the expression of Foxo1, in Treg cells, SGK1 deletion can prevent Foxo1 from nucleating, thereby promoting binding of Foxo1 to the Foxp3 CNS1 region, increasing the expression of Foxp3, promoting the differentiation of Treg cells and inhibiting the development of Th17 cells. Retinol-induced death-related gene (GRIM) 19 reduces Th17 differentiation and p-STAT3 expression, upregulates p-STAT5 expression, and enhances Treg differentiation. Inhibition of casein kinase 2 (CK2) inhibits the differentiation of Th17 cells and induces the differentiation of Tregs by blocking STAT3 phosphorylation and the mTOR signalling pathway (61). Ammonium trichlorotellururate compound AS101, as a small non-toxic immunomodulator, can not only reduce the immune response of pathogenic Th17 cells, but also promote the differentiation of Treg cells without relying on TGF-β (62). Zhu et al. confirmed that insulin binding protein-5 (IGFBP5) can reduce the percentage of Th17 cells and increase the percentage of Treg cells by inhibiting the expression of the pro-inflammatory cytokines TNF-α, IL-1β, and IFN-γ and altering the ratio of Th17/Treg (63).

**Figure 4:**
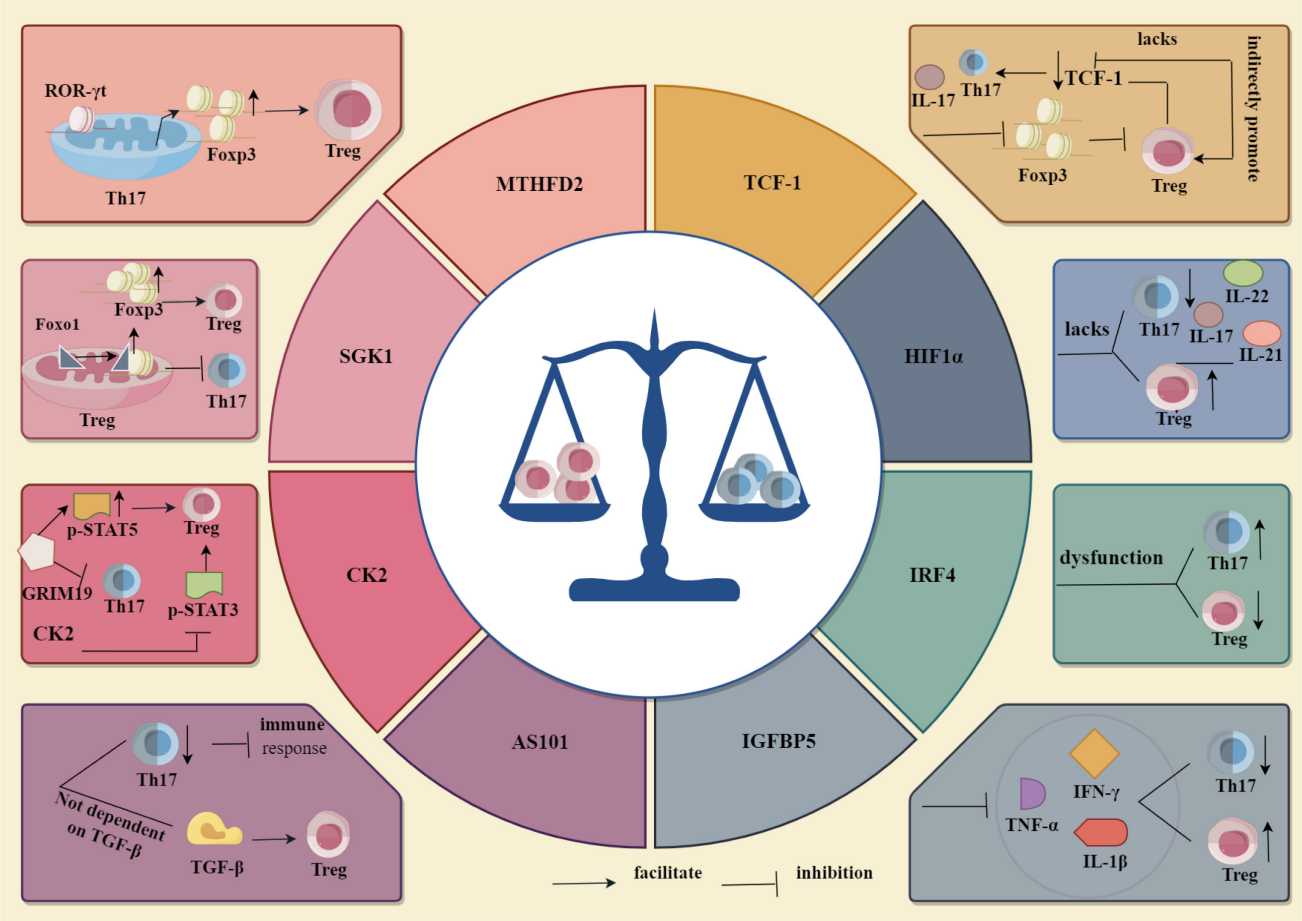
Regulation of Th17, Treg differentiation by cytokines and transcription factors. Among cytokines MTHFD2, SGK1, CK2, AS101 and IGFBP5, through their respective mechanisms of action, MTHFD2 and SGK1 promoted Th17 cell differentiation and inhibited Treg cell differentiation, whereas CK2, AS101 and IGFBP5 inhibited Th17 cell differentiation and promoted Treg cell differentiation. Whereas among the transcription factors IRF4, HIF1α and TCF-1, IRF4 inhibited Th17 cell differentiation and promoted Treg cell differentiation through its related mechanism, HIF1α and TCF-1 were opposite to IRF4, i.e., they promoted Th17 cell differentiation and inhibited Treg cell differentiation.

In addition to cytokines, transcription factors play an important role in the differentiation of Th17 and Treg cells. For example, dysfunction of the transcription factor IRF4 affects the ratio of Th17 to Treg cells and leads to the dysfunction of Treg cells (64). In addition, the upregulated protein and gene expression of transcription factor hypoxia inducible factor 1 (HIF1α) leads to the differentiation of Th17 cells, while the downregulated protein and gene expression of HIF1 α leads to the decreased expression of Th17 family cytokines IL-17, IL-21, and IL-22 and enhances the differentiation of Treg cells (65). Transcription factor TCF-1 inhibits the development and function of Treg cells by inhibiting the expression of Foxp3, while Treg cells can also reduce the expression of TCF-1 and increase the signal transduction of Th17 and IL-17. In addition, although TCF-1 deficiency does not change the transcriptional characteristics of Treg cells, it activates alternative signalling pathways, thus promoting the differentiation of Treg cells (**Figure 4**) (66).

### 2.2 Regulation of signal pathway on Th17 and Treg differentiation

**Figure 5:**
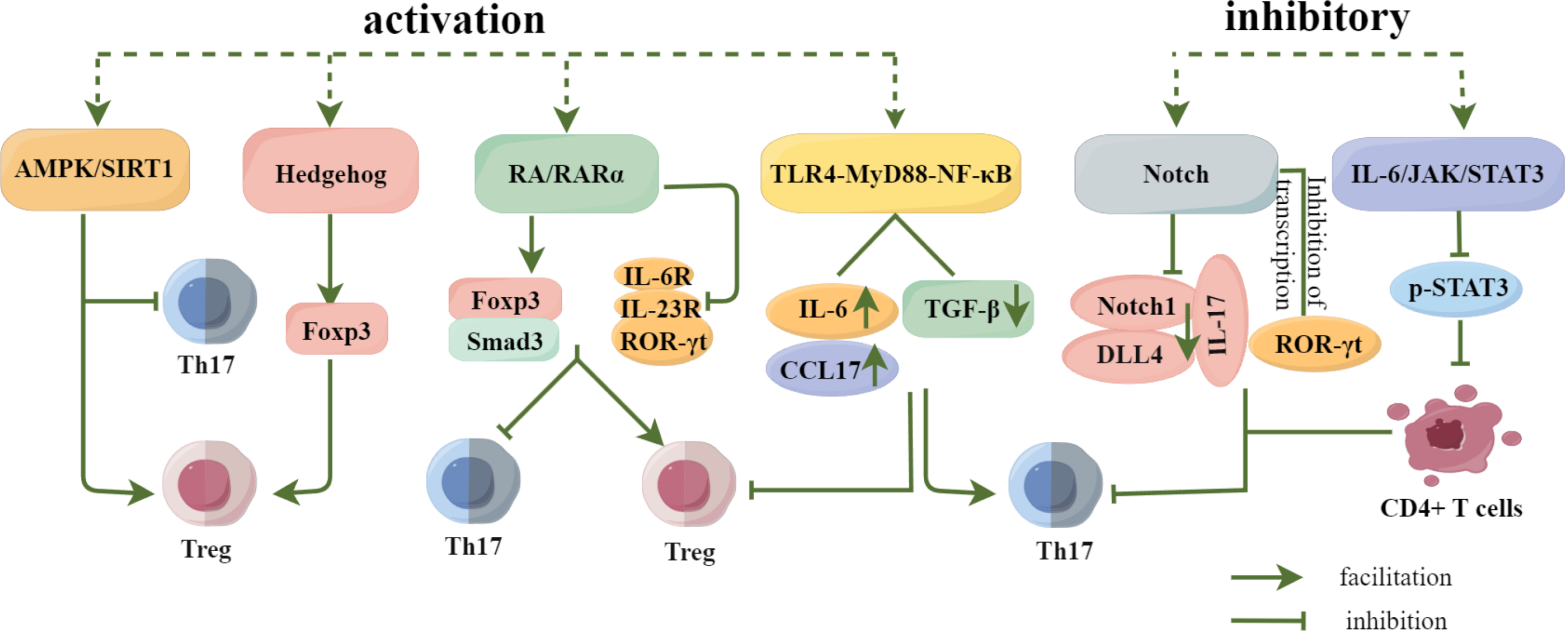
Regulation of Th17 and Treg differentiation by signaling pathways. Activation of the AMPK/SIRT1 and Hedgehog signaling pathways promoted Treg cell differentiation, and activation of the RA/RARα signaling pathway not only promoted Treg cell differentiation, but also inhibited Th17 cell differentiation.Activation of the TLR4-MyD88-NF-κB pathway inhibited Th17 cell differentiation while promoting Treg cell differentiation, whereas Inhibition of Notch and IL-6/JAK/STAT3 signaling pathways significantly reduced or inhibited Th17 cell differentiation and regulated Th17/Treg homeostasis.

In addition to inflammatory factors, cytokines, and transcription factors, the activation and inhibition of signalling pathways also play an important role in the differentiation of Th17 and Treg cells. (**Figure 5**). For example, Ma et al. (67) found that the activation of the AMPK/SIRT1 signalling pathway not only blocks the differentiation of pathogenic Th17 cells but also enhances the production of protective Treg cells. Additionally, the Hh signalling pathway promotes Treg differentiation by upregulating the expression of transcription factor Foxp3. Inhibition of the Hh signalling pathway suppresses the immunosuppressive activity of Treg cells and promotes the transformation of Tregs to Th17 cells (68). Xiao et al. (69) confirmed that promoting RA/RAR α signalling pathway can upregulate Smad3 and Foxp3 expressions, promote Treg differentiation, and inhibit Th17 differentiation by inhibiting the expression of IL-6R and IL-23R and the production of ROR-γt. Activation of the TLR4-MyD88-NF-κB signalling pathway can significantly increase the expression of IL-6 and CCL17 and significantly decrease the expression of TGF-β, resulting in the increase of Th17 cells and the decrease of Treg cells (70). Studies have found that inhibiting the activation of Notch signalling pathway can effectively reduce the response of Th17 cells, down-regulate the expression of Notch1, DLL4, IL-17 and the transcription of ROR γt, reduce the level of Th17 cells, downregulate Notch1, DLL4, and IL-17 expressions and ROR γt transcription, reduce the level of Th17 cells, and effectively restore the balance of Th17/Treg (71, 72). In addition, inhibiting the expression of p-STAT3 in the IL-6/JAK/STAT3 signalling pathway can significantly inhibit the differentiation of CD4+T cells into Th17 cells, downregulate the secretion of IL-17A, and effectively regulate the balance between Th17 and Treg cells (73). Therefore, the differentiation of Th17 and Treg cells is affected by many factors, and the regulation of Th17 and Treg cell differentiation and the balance of Th17/Treg should be investigated from many angles.

## 3 Relationship between Th17/Treg imbalance and rheumatoid arthritis and postmenopausal osteoporosis

In recent years, bone immune regulation has become a hot topic in research on bone metabolic diseases (74). Research has found that bone immunity has a destructive effect (75), and bone immune disorders induced by Th17 cells, Tregs, and related cytokines are important triggers for the development of RA and PMOP (17, 76) Many studies have shown that the proportion of Th17 cells in the serum and synovium of patients with RA and collagen-induced arthritis (CIA) rats is far higher than that of Treg cells, however, inhibiting Th17 cells and upregulating Treg cells can alleviate arthritis and bone destruction (77, 78), indicating that regulating the Th17/Treg cell balance may be important for the treatment of RA. In addition, some studies have confirmed that in patients with PMOP and ovariectomized (OVX) mice, Th17 cells and their related cytokines, such as IL-17, TNF-α, and IL-6, increased, Treg cells and their related anti-inflammatory factors decreased, OC-mediated bone resorption and OB-mediated bone formation were out of balance, the Th17/Treg ratio increased, and the bone remodelling process was worsened. By inhibiting the differentiation of Th17 cells and promoting the differentiation of Tregs, this balance was restored and bone remodelling was effectively regulated (35, 38). These results indicated that the Th17/Treg balance is closely related to the prevention and treatment of PMOP.

### 3.1 Mechanism of Th17/Treg imbalance in rheumatoid arthritis

As one of the most common autoimmune diseases, RA is characterised by chronic arthritis, cartilage degeneration, and local and systemic bone loss (79). Th17 cells have been shown to promote the production of matrix metalloproteinases and the influx of immune cells by secreting inflammatory cytokines, such as IL-17, IL-21, TNF-α, and IL-6, and aggravate joint destruction while maintaining inflammatory reaction (34, 80, 81). In addition, the synergistic effect of IL-17 and TNF-α can lead to cartilage destruction and matrix metalloproteinase release, which further promote the degradation of cartilage matrix and accelerate the progress of RA (82). In contrast, IL-17, the main cytokine secreted by Th17 cells, not only directly upregulates the expression of RANKL in synovial fibroblasts of patients with RA but also promotes the differentiation of Th17 cells by upregulating the expression of prostaglandin E2 (PGE2) in OBs, thus inducing OC production and joint destruction (20, 83). IL-21 not only relies on RANKL to promote OC production in RA but also directly promotes OC production through the PI3K/AKT signalling pathway, which is independent of RANKL (84). TNF-α and IL-6, as OC-promoting factors, aggravate the inflammatory reaction of patients with RA and accelerate bone destruction (85).

Contrary to Th17 cells, Treg cells expressing transcription factor Foxp3 can not only inhibit the differentiation of Th17 cells by secreting anti-inflammatory cytokines IL-10, IL-4, IL-35, and TGF-β, but also inhibit inflammatory reactions and bone resorption (86, 87). IL-10 is an important anti-inflammatory and immunosuppressive cytokine, reduces the expression of serum IL-6 by upregulating suppressor of cytokine signalling 1 (SOCS1), which not only leads to a decrease in anti-type II collagen antibody levels but also directly acts on various immune cells and inhibits T cells from producing pro-inflammatory cytokines, thus reducing the severity of arthritis and playing a key protective role in RA (88). In addition, IL-10 and IL-4 not only upregulate osteoprotegerin (OPG) and downregulate RANKL and RANK (89), but also promote M2 macrophage polarization, weaken macrophage differentiation into OCs, promote OB proliferation and osteogenic differentiation of BMSCs, and inhibit bone destruction (90). IL-35, a newly discovered anti-inflammatory cytokine, plays an immunosuppressive role by enhancing the expression of Treg cells in patients with RA and protecting against RA (91). As a widely used immunosuppressant, TGF-β can not only regulate the proliferation, differentiation, and biological function of various immunoreactive cells, but also stimulate the proliferation and differentiation of BMSCs, promote the proliferation of OBs and chondroblasts and the synthesis of extracellular matrix, and inhibit the production and biological activity of OCs (92, 93). In contrast, Tregs inhibit bone resorption via cell-to-cell contact through cytotoxic T lymphocyte antigen-4 (CTLA4) (94). CTLA-4 is typically regarded as a marker of Tregs. By binding to CD80/CD86 on the OC precursor, CTLA-4 activates indoleamine 2, 3-oxygenase 2 (IDO) in OCPs. Activated indoleamine 2, 3-dioxygenase can degrade tryptophan, promote the apoptosis of OC precursor cells, and inhibit bone resorption (95, 96).

Therefore, Th17 cells secrete inflammatory factors related to RA, which not only promote and maintain inflammatory reactions but also promote the degradation of the cartilage matrix, the differentiation and maturation of OC, and accelerate joint destruction in RA. Treg cells secrete anti-inflammatory cytokines that inhibit inflammatory reactions, promote OB proliferation, weaken OC differentiation, and play a role in inhibit bone resorption in RA, indicating that an imbalance in the Th17/Treg ratio is closely related to the occurrence and development of RA.

### 3.2 Mechanism of Th17/Treg imbalance in postmenopausal osteoporosis

Osteoporosis (OP) is a common systemic bone disease mainly caused by the uncoupling of bone formation and resorption during bone remodelling (97). PMOP is a type of high bone turnover OP caused by a sudden decrease in oestrogen levels in postmenopausal women (98).

Oestrogen is an important regulator of bone homeostasis and plays a role through two receptors, namely, ERα and ERβ, although ERα is more important in the regulation of bone metabolism. Oestrogen can induce the transcription of Fas ligand (FasL) in OBs by binding with ERα and activate the Fas/FasL pathway in OCs. Moreover, Oestrogen increased the transcription of matrix metalloproteinase-3 (MMP-3) and FasL divided from the cell surface by MMP-3 to form soluble FasL, thus inducing osteoclast Apoptosis (99). In addition, oestrogen can promote the proliferation and differentiation of mesenchymal stem cells into OB precursor cells by binding with ERα, thereby increasing OB activity. Thus, oestrogen not only promotes OB activity but also prevents the formation of OCs, thereby regulate bone remodelling (29).

In addition, studies have found that the immune system plays an important role in bone remodelling (100), and that T cells are the main body of immune regulation. Studies have confirmed that oestrogen receptors are present on T cells and that changes in oestrogen levels can directly affect T cell proliferation and activation (101). For example, oestrogen can induce the expression of FOXP3 in Treg cells and stimulate the activity and secretion of IL-10 and TGF-β while attenuating the secretion of IL-17 and RANKL in Th17 cells (102), IL-10 not only inhibits the proliferation and production of other cytokines, but also upregulates the secretion ofOPG and downregulates the expression of RANKL and M-CSF to inhibit the differentiation and maturation of OC (103, 104). TGF-β promotes the survival, osteogenic differentiation and migration of OBs through the PI3K/AKT/mTOR/S6 kinase 1 signalling pathway (105). In addition, evidence has shown that Treg cells induce BMSC to differentiate into OB by secreting TGF-β and activating intracellular effectors, such as mitogen-activated protein kinase (MAPK) and Smad-related proteins, and promote OB proliferation and differentiation (106).

Studies have also shown that Treg cells not only inhibited OC differentiation and bone resorption but also promoted OB survival. In addition, Treg cells can promote the binding of the surface molecule CTLA-4 withCD80/CD86 expressed on OC precursor cells, which leads to the activation of indoleamine-2,3-dioxygenase and degradation of tryptophan and promotes the apoptosis of OC precursor cells, thus inhibiting bone resorption (95, 96), These findings indicate that oestrogen plays an important regulatory role in bone remodelling by regulating the expression of Treg cells.

Under oestrogen deficiency, Th17 cells not only directly express RANKL to promote OC production, but also secrete a large number of inflammatory factors, such as IL-17, TNF-α and IFN-γ. IL-17 and TNF-α indirectly promote OC production by inducing human bone marrow mesenchymal stem cells to secrete M-CSF and RANKL (38, 107), TNF-α can improve OC production by coordinating with RANKL and increasing RANK expression of OC precursor (33), and also indirectly stimulate OC development and function by reducing OPG release from OBs (108), indicating that Th17 cells also indirectly increase OC by reducing OPG/RANKL ratio. In addition, IFN-γ can also promote monocyte fusion and bone resorption by inducing T cells to produce RANKL and TNF-α in the late stage of OC formation (109), which indicates that oestrogen and T cells are closely associated with bone remodelling. Therefore, regulating bone remodelling by regulating the imbalance of Th17/Treg cells can provide a new method for the treatment of PMOP.

The above findings indicate that the pathogenesis of RA and PMOP is related to the imbalance of Th17/Treg cells; however, the factors and mechanisms that promote an imbalance of Th17/Treg to induce RA and PMOP, which are related to inflammatory reactions and bone remodelling disorders, are not completely consistent, as shown in **Table 1**.

**Table 1:** Association of Th17/Treg imbalance with rheumatoid arthritis and postmenopausal osteoporosis.

		RA		PMOP	
		Th17	Treg	Th17	Treg
consistent	Inflammation response	1.Secretion of IL-17, IL-21, TNF-α, and IL-6 exerts a pro-inflammatory effect that promotes the production of MMP and the influx of immune cells, exacerbating joint destruction while maintaining the inflammatory response 2.TNF-α and IL-6 accelerate inflammatory response while exacerbating the bone destruction in RA	1.Secretion of IL-10, IL-4, IL-35 and TGF-β inhibits Th17 cell differentiation, which in turn inhibits inflammatory response and bone resorption 2. IL-10 upregulates SOCS1, reduces IL-6 expression, leads to a decrease in anti type II collagen antibody levels, and inhibits the production of pro-inflammatory factors	Secretion of IL-17, TNF-α and IFN-γ exerts pro-inflammatory effects	Secreting IL-10 and TGF- β Exert anti-inflammatory effects
	Bone resorption or bone formation	1.IL-17 directly upregulated RANKL expression on synovial fibroblasts and PGE2 expression in OB, which promoted Th17 differentiation and induced OC generation, leading to joint destruction 2.IL-21 promotes OC production by relying on RANKL and PI3K/AKT signaling pathways	1.IL-10 and IL-4 upregulate OPG, decrease RANKL and RANK expression, promote M2-type macrophage polarization changes, attenuate macrophage differentiation to OC, promote OB proliferation and osteogenic differentiation of BMSC, and inhibit bone destruction 2.TGF-β promotes OB and chondrocyte proliferation and extracellular matrix synthesis, and inhibits OC production and bioactivity 3.Inhibition of bone resorption through cell-cell contact through CTLA4	1.Direct expression of RANKL promotes OC generation 2.IL-17 and TNF-α promote OC production by inducing hBMSC to secrete M-CSF and RANKL 3.TNF-α on the one hand improves OC production by coordinating with RANKL and increasing RANK expression in OC precursors. On the other hand TNF-α indirectly stimulates OC development and function by decreasing osteoblast OPG release 4.IFN-γ promotes monocyte fusion and bone resorption in late OC by inducing T cells to produce RANKL and TNF-α	1.IL-10 inhibited T cell proliferation, up-regulated OPG and down-regulated RANKL and M-CSF, and suppressed OC differentiation and maturation 2.TGF-β, on the other hand, promotes OB survival, differentiation and migration through the PI3K/AKT/mTOR/S6 kinase 1 signaling pathway 3.Secretion of TGF-β and activation of MAPK and Smad-related proteins induced BMSC differentiation into OB and promoted OB proliferation and differentiation 4.Inhibition of bone resorption through cell-cell contact through CTLA4
discrepancy		IL-17 synergizes with TNF-α, leading to cartilage destruction and MMP release, which promotes cartilage matrix degradation	IL-35 upregulates Treg and exerts immunosuppressive effects	/	/

## 4 Immunotherapy for rheumatoid arthritis and postmenopausal osteoporosis by regulating Th17/Treg balance

As a dynamic organ, bone is composed of OBs, OCs, and other basic elements that are constantly removed throughout the body to maintain bone calcium metabolism, bone biomechanical function, and good bone structure (110, 111). The immune system protects the body from external antigens. Systematic studies have shown a close relationship between the immune and skeletal systems, they share various regulatory molecules, including cytokines and transcription factors. The physiological and pathological state of one system inevitably affects the other, based on an “immune-bone remodelling” regulatory network, which plays a complex and delicate role in bone remodelling (100, 112, 113). During “immune bone remodelling”, an imbalances in the regulatory network lead to various chronic bone diseases, such as RA and PMOP (114). Chronic inflammation and immune abnormalities are the primary causes of RA (115, 116). T cells are important components of the immune system, which not only play a role not only in the immune system but also in bone remodelling (117). Th17 and Treg cells, which initially differentiate from CD4+T cells, are considered the dominant T cells that regulate RA progression (118). Th17 cells mediate inflammation, pannus growth, OC formation, and synovial neovascularization during the development of RA (119, 120), while Treg cells can inhibit the function of Th17 cells during the development of RA (121), which indicates that Th17 and Treg cells are the main driving factors regulating the immune response during the development of RA. In addition, some studies have confirmed that oestrogen is involved in immune-bone remodelling, In chronic inflammation caused by oestrogen deficiency, activated T cells lead to an increase in pro-inflammatory cytokines, the osteogenic differentiation ability of BMSCs is damaged, Th17 cells are significantly increased, Treg cells are significantly reduced, OCs are increased, bone resorption is enhanced, and the balance of Th17/Treg cells is disordered (28, 122, 123). Therefore, regulating the imbalance between of Th17/Treg cells may be the most direct and main factor in for preventing RA and PMOP.

### 4.1 Conventional Western medicine treatments

Currently, the drugs used to treat RA clinically are disease-modifying antirheumatic drugs (DMARDs), including routinely synthesised DMARD (methotrexate, leflunomide, sulfasalazine), biological (b)DMARDs (tumour necrosis factor inhibitors infliximab, etanercept, adalimumab, golimumab, etc.), and targeted synthetic of (ts)DMARDs, (Janus kinase inhibitors tofatinib, barisitinib, pefitinib, figotinib, and upatinib) (124).

Methotrexate (MTX) is a folate-resistant metabolite that inhibits DNA synthesis, repair, and cell replication; it can not only optimise biological DMARD but also has a lower dose, lower price, and convenient administration compared with other traditional synthetic DMARD recognised as the first-choice DMARD in RA management (125). However, its main side effects include gastrointestinal diseases, liver disorders, pneumonia, haematological diseases, infections, nephrotoxicity, and dermatitis. Gastrointestinal side effects are the most common side effects of MTX, whereas haematopoiesis, carcinogenicity, and hepatotoxicity are recognised as toxic reactions (126). Leflunomide (LEF), a new first-line immunosuppressant for RA, has no significant curative effect compared to MTX, but it can reduce the damage of rheumatism to the joint bone by inhibiting joint OC synthesis, thus significantly improving the joint function and quality of life of patients (127). In addition, the combination of LEF and MTX can double inhibit the synthesis of dihydrofolic acid, maximise the curative effect of RA treatment without an obvious increase in side effects, and has the potential to treat other immune diseases (128). However, LEF is more expensive than MTX and can cause adverse gastrointestinal reactions, such as nausea, vomiting, diarrhoea, elevated transaminase, itching, and rash, which limit its clinical use (129, 130). Sulfasalazine(SSZ) is a recognised DMARD used to treat RA; however, its exact mechanism has not been fully elucidated (131). at present, SSZ is believed to play an antirheumatic role mainly through antibacterial, anti-inflammatory, and immune-regulatory mechanisms (132). SSZ is usually well tolerated in clinical Trials and presents a similar clinical efficacy as MTX and LEF; however, it has dual anti-inflammatory and antibacterial effects, improves the articular and extra-articular manifestations of patients with RA, and is safe during pregnancy and lactation; therefore, it is generally considered as one of the more effective traditional DMARD. SSZ is primarily used as the initial treatment for RA in the clinic (133, 134); however, the most common adverse reactions are nausea, vomiting, diarrhoea, anorexia, headache, dizziness depression, rash, and bone marrow suppression, which limit its clinical application (135).

In recent decades, biological agents have been identified that block cytokines on a large scale and significantly improve the joint function and quality of life of patients with RA (136). TNF-α inhibitors, as a kind of biological agents, are the initial treatment choices for patients who require need biological agents, and they mainly include infliximab, etanercept, adalimumab,and golimumumaband (137). TNF-α is related to systemic inflammation and acute phase reaction. Infliximab, as a chimeric monoclonal antibody that inhibits cytokine activation of TNF receptor complex, shows high affinity for TNF-α, and can reduce a series of complications related to TNF-α, including systemic inflammation, increase adhesion molecules, induce pro-inflammatory cytokines, increase leukocyte migration to tissues, and diffuse the activation of acute phase reactions. It has a remarkable curative effect in maintaining joint function and improving disease progression and activity (138, 139). Etanercept is one of the biological agents that have revolutionized the treatment of RA in recent years, and it is composed of the extracellular portion of the TNF-α receptor and Fc portion of immunoglobulin G (IgG), Moreover, it is well tolerated and has a low incidence of serious adverse events. Except for a shorter half-life than infliximab, there was no other significant difference was observed between etanercept and infliximab, and the most common adverse reactions are injection site reactions, such as redness, swelling, pain, upper respiratory tract infection, and headache (140, 141). Adalimumab is a mono-clonal antibody of recombinant IgG, and it can inhibit cytokine-related inflammatory process and has low immunogenicity potential (142). It can protect the joint function of patients mainly by activating NF-κB receptor of stromal cells and OBs and blocking the destruction of bone and cartilage, and the most common adverse reactions are infection and injection-site reactions (143). GolimumabIs is a monoclonal antibody that can bind soluble and transmembrane TNF, thereby blocking the binding and activity of TNF-α receptor (144), A previous study found that after stopping the previously used TNF-α antagonist, the use of golimumab improved the signs, symptoms, and physical functions, mainly by improving the cardiovascular system (CVS) and endothelial function by reducing arterial wall hardness and atherosclerosis (145); however, the cost is high, and the risk of infection is high. Further, it induces central nervous system symptoms such as headache and dizziness, and injection site reactions.

JAKs are non-receptor tyrosine kinases involved in the activation of inflammatory cascades in immune cells, including JAK1, JAK2, JAK3, and tyrosine kinase (TYK), which play important roles in cytokine signalling. For example, the combination of cytokines and their receptors leads to the phosphorylation of JAK, whereas p-JAK activates signal transducers and transcription activators (STAT), which dimerise and transpose to the nucleus. Members of the STAT family act as transcription factors that regulate the transcription of target genes (146, 147). JAK kinase inhibitors (JAKis) are a new class of orally targeted drugs for the treatment of RA that may prevent RA attacks in patients with undifferentiated arthritis by inhibiting the STAT4 signalling pathway (148). To date, five JAK inhibitors have been approved for the treatment of RA: tofatinib, barektinib, upatinib, fegotinib, and peifitinib (149). Tofatinib is a small-molecule compound that is convenient for oral administration and can inhibit all JAKs except TYK2. However, in clinical trials, upper respiratory tract infections, headaches, diarrhoea, memory loss, and nasal inflammation were observed (150, 151). Barektinib targets JAK1 and JAK2, which is effective for relieving pain; however, there are risks associated with its use, such as infection, thrombosis, leukopenia, elevated cholesterol levels, and lymphoma (152). Both upatinib and fegotinib specifically target JAK1, among which upatinib is more effective than a TNF inhibitor in RA treatment, and fegotinib has the highest selectivity for JAK1 is convenient to take orally, and has a lower probability of adverse events compared with upatinib. However, upatinib causes upper respiratory tract infection, gastrointestinal discomfort, acne, headache, and decreased white blood cell counts, while fegotinib treatment causes adverse reactions, such as infection, gastrointestinal discomfort, liver function damage, depression, and insomnia (153, 154). In addition, peffitinib, a multi-target inhibitor, showed the highest selectivity for JAK3, however, it is expensive, and has common adverse reactions, such as infection, tumours, venous thromboembolism, and hyperlipidaemia (155). Thus, the side effects of JAK inhibitors limiting their clinical application, and their high price exerts a heavy economic burden on patients with RA, which leads to patients with RA not insisting on or hesitating to use JAK inhibitors for treatment (156).

In addition, although DMARDs are considered the first choice for RA treatment, non-steroidal anti-inflammatory drugs (NSAIDs) (e.g. ibuprofen and naproxen sodium) play strong anti-inflammatory and immunomodulatory roles in RA by inhibiting cyclooxygenase activity and reducing prostaglandin synthesis. NSAIDs can also reduce the monocyte-macrophages number in the circulatory system, inhibit inflammatory factors and prostaglandins synthesis, prevent inflammatory cell exudation, inhibition of OC production, and reduce articular cartilage destruction; thus, they have been widely used in RA treatment. However, gastrointestinal irritation, cardiovascular diseases, liver and kidney injuries, and allergic reactions can occur during treatment (157, 158). Glucocorticoids (cortisone, prednisone, and dexamethasone) inhibit the infiltration, exudation, and production of inflammatory factors by reducing capillary permeability and binding to glucocorticoid receptors. In addition, glucocorticoids interfere with and block lymphocyte recognition by inhibiting antibody reactions, macrophage phagocytosis, and antigen processing, and regulating the number and distribution of lymphocytes, which play anti-inflammatory and immune roles, relieve joint inflammation and pain, and delay joint injury (159). However, long-term application of hormonal drugs has side effects, such as increased blood sugar, gastric ulcers, osteoporosis, and insomnia. Therefore, glucocorticoids should be used in small doses over a short course for the clinical treatment of RA, and DMARDs must be used simultaneously (160).

For PMOP, there are mainly bone resorption inhibitors (bisphosphates, oestrogens, and calcitonins) and bone formation promoters (parathyroid hormone and its analogues, including teripatide and abaloparatide) are available (161). Among them, pyrophosphate analogues in bisphosphates combine with hydroxyapatite crystals in the bone, inhibit the function and recruitment of OCs, and increase the apoptosis of OCs, thereby effectively reducing the risk of spinal, non-spinal, and hip fractures. Therefore, they are used as the first-line treatment drugs in most patients with an increased risk of postmenopausal fractures. However, bisphosphates are poorly absorbed when administered orally. Therefore, oral administration with water needs to be performed on an empty stomach in the morning, with water 30-60 minutes before eating, and patients need to remain upright to avoid irritating the oesophagus. In addition, oral bisphosphate can cause adverse reactions, such as musculoskeletal pain, gastrointestinal irritation, oesophageal reflux, and ulcers (162, 163). Oestrogen deficiency is one of the main causes of PMOP. Oestrogen not only directly acts on bone cells by binding to oestrogen receptors but also indirectly regulates immune cells and immune factors, thus promoting OB proliferation and differentiation, inducing OC apoptosis, and inhibiting immune activity, thereby maintaining the balance between bone resorption and bone formation and protecting bone tissue. However, the side effects of oestrogen, such as cardiovascular events, thromboembolic diseases, and breast cancer, make it difficult to determine the balance between its risks and benefits (164, 165). Calcitonin, as the most useful substitute drug after acute spinal fracture, mainly binds to the OC membrane surface receptor, activates adenylate cyclase to increase cyclic adenosine monophosphate (cAMP), activates the phospholipid inositol system to increase cytoplasmic free calcium, inhibits OC absorption, promotes OB synthesis, and increases bone mass. Moreover, it has an obvious analgesic effect, and is a mild regulatory drug for treating PMOP. However, it still causes facial flushing, fever, headache, dizziness, nausea, vomiting, anorexia, rash, and other adverse reactions (166).

Parathyroid hormone and its analogues teriparatide and abaloparatide, which promotes bone formation, are anabolic agents that have been approved for use in PMOP (167). Teriparatide mediates bone metabolism by inhibiting OB apoptosis, activating bone-lining cells, and enhancing OB differentiation, which can reduce the incidence of fractures in postmenopausal women, and significantly reduce the risk of recurrent vertebral fractures (168). Abaloparatide is a selective agonist of the parathyroid receptor 1 (PTHR1) and can combine with PTHR1 to activate the cyclic adenosine monophosphate (cAMP) signalling pathway in target cells, thereby regulating metabolism and promoting bone formation. Compared to teriparatide, it can significantly reduce bone absorption and promote bone formation; therefore, it is often used in patients with a high fracture risk or previous osteoporosis treatment failure or intolerance (169). However, the study also found that side effects, such as nausea, dizziness, headache, palpitation, liver damage and increased osteosarcoma risk, will occur when treating PMOP with teriparatide and abalopatide; therefore, it is necessary to limit their use in clinical applications (170). Therefore, although Western medicine treatments for RA and PMOP have achieved certain clinical efficacy, they have many side effects, high costs, and complex administration methods. as described in **Table 2**. Therefore, there is an urgent need to develop new drugs for the prevention and treatment of RA and PMOP that are safe, low-cost, attenuated, synergistic, simple, and easy to use. Thus, the targeted regulation of Th17/Treg cell balance has become an effective strategy for the prevention and treatment of RA and PMOP.

**Table 2:** Advantages and shortcomings of conventional western drug for RA and PMOP.

Drug type	Medicines	Advantages	Shortcomings	Diseases	Refs
csDMARDs	Methotrexate (MTX)	Lower dosage, lower price, easy to take	Gastrointestinal diseases, liver disorders, pneumonia, hematological diseases, infections, nephrotoxicity, dermatitis, etc	RA	140-141
	Leflunomide (LEF)	Combination with MTX in RA treatment maximizes efficacy without significantly increasing side effects, and has potential for treating other immune disorders	Expensive, nausea, vomiting, diarrhea, elevated aminotransferases, itching, rash, etc	RA	143-145
	Sulfasalazine (SSZ)	Well tolerated, broadly similar clinical efficacy to MTX, leflunomide, etc., dual anti-inflammatory and antimicrobial effects, improvement of both joint and extra-articular manifestations in patients with RA, safety in pregnancy, pregnancy and lactation	Gastrointestinal reactions, headache, dizziness, rash and bone marrow suppression	RA	148-150
bDMARDs -TNF-α inhibitors	Infliximab	Inhibits systemic inflammation, increases adhesion molecules, and is effective in maintaining joint function and improving disease activity	Expensive, no significant repair of already damaged joints, risk of chronic inflammatory skin disease, congestive heart failure, and lymphoma	RA	153-154
	Etanercept	Well tolerated with low incidence of serious adverse events	Injection site reactions such as redness, swelling and pain as well as upper respiratory tract infections and headaches	RA	155-156
	Adalimumab	Inhibits cytokine-associated inflammatory processes with low immunogenicity potential, blocking bone and cartilage destruction and exerting joint protective functions	Upper respiratory tract infections, nasal inflammation and injection site reactions, etc	RA	157-158
	Gollimumab	The use of golimumab in conjunction with discontinuation of previously used TNF-α antagonists resulted in sustained improvements in signs, symptoms, and physical functioning in patients with RA, primarily including improvements in the cardiovascular system and endothelial functioning, and reductions in arterial wall stiffness and atherosclerosis	Expensive, high risk of infection, and central nervous system symptoms such as headache, dizziness, and injection site reactions	RA	160
tsDMARDs -Janus kinase inhibitor	Tofacitinib	Convenient oral administration, can inhibit all JAKs except TYK2, and the efficacy is exact	Expensive, presence of upper respiratory infections, nasal inflammation, headaches, diarrhea, etc	RA	165-166
	Baricitinib	Highly effective in reducing pain	Infections, thrombosis, leukopenia, elevated cholesterol levels, and increased risk of lymphoma	RA	167
	Upadacitinib	More effective than TNF inhibitors in the treatment of RA	Expensive, upper respiratory tract infections, gastrointestinal distress, headaches, lowered white blood cell counts, etc	RA	168
	Filgotinib	Easy to administer orally, highest selectivity for JAK1, lower probability of adverse events than upatinib	Expensive, infections, gastrointestinal distress, liver impairment, depression, insomnia, etc	RA	169
	Peficitinib	Highest selectivity for JAK3	Expensive, infections, tumors, venous thromboembolism and hyperlipidemia	RA	170
nonsteroidal anti-inflammatory drug (NSAID)	Ibuprofen	Relieve pain and inflammation	Gastrointestinal irritation, cardiovascular system disorders, hepatic and renal injuries, allergic reactions, etc	RA	172-173
	Naproxen Sodium				
Glucocorticoids	Cortisone	Immunomodulatory and anti-inflammatory effects, reducing inflammation and pain and slowing down joint damage	Elevated blood sugar, gastric ulcers, induced osteoporosis, insomnia, etc	RA	174-175
	Prednisone				
	Dexamethasone				
Bone resorption inhibitor	Bisphosphonates	Inhibition of OC function and recruitment and increase in OC apoptosis may effectively reduce the risk of vertebral, non-vertebral and hip fractures	Oral malabsorption, adverse reactions such as musculoskeletal pain, gastrointestinal irritation, esophageal reflux and ulcers	PMOP	177-178
	Estrogen	Promotes OB proliferation and differentiation, induces OC apoptosis, inhibits immunoreactivity, and thus maintains the balance between bone resorption and bone formation and protects bone tissue	Triggers cardiovascular events, thromboembolic disease, breast cancer, etc	PMOP	179-180
	Calcitonin	Inhibits OC resorption, promotes OB synthesis, has increased bone mass and significant analgesic effect, mild action	Facial flushing, fever, headache, dizziness, nausea, vomiting, poor appetite, rash, etc	PMOP	181
Bone formation enhancers - parathyroid hormone and its analogs	Teriparatide	Inhibition of OB apoptosis, activation of bone-lining cells, and enhancement of OB differentiation reduces the incidence of fractures and significantly decreases the risk of vertebral re-fracture in postmenopausal women	Side effects such as nausea, dizziness, headache, heart palpitations, increased risk of osteosarcoma	PMOP	183,185
	Abaloparatide	Significantly reduces bone resorption and promotes bone formation, and is commonly used in patients at high risk of fracture or who have failed or are intolerant to prior OP therapy		PMOP	154-185

### 4.2 Probiotics

Probiotics are active microorganisms that have been shown to have beneficial effects on many diseases (171). Studies have found that probiotics have a variety of immunomodulatory characteristics, which can increase the strength of the intestinal epithelium and play a role in bone protection by controlling intestinal microflora to reduce antigen presentation and activation of intestinal immune cells; therefore, the “intestine-immunity-bone” axis is affected by probiotics and has attracted wide attention from researchers worldwide (172). Recent studies have found that probiotics can play a therapeutic role in bone diseases, such as RA and PMOP, by regulating the Th17/Treg cell balance (173, 174). The results are summarised in **Table 3**.

**Table 3:** Therapeutic effects of probiotics on RA and PMOP by modulating Th17/Treg balance.

Probiotics	Reduced immune inflammatory factors	Anti-inflammatory factors	Disease	Refs
Lactobacillus casei CCFM1074	IL-17, IL-1β, IL-6, TNF-α, Th17	IL-10, Treg	RA	191
lactobacillus rhamnosus	IL-6, IL-17, TNF-α, RANKL, Th17, CD4+RoR-γt+Th17	IL-4, IL-10, IFN-γ, Treg, CD8/CD4+Foxp3+ Treg	PMOP	193
Lactobacillus rhamnosus GG	TNF-α, IL-17, RANKL, RoRγt, Th17	TGF-β, IL-10,Foxp3, Treg	PMOP	194
bifidobacterium longum	TNF-α, IL-6, IL-17, Th17	IFN-γ, IL-10, Bregs, Treg	PMOP	196

*Lactobacillus* is a widespread probiotic that thrives in the acidic intestinal tract with the support of glucose in the stomach (175). Fan et al. (176) performed an experimental study and found that *Lactobacillus casei* can inhibit the development of RA in rats by changing the intestinal microbiome, inhibiting the levels of IL-17, IL-1β, IL-6, and TNF-α in inflammatory cells, and changing the ratio of Th1/Th17. In addition, a further study revealed that in CIA rats, the levels of IL-1β, IL-6, and TNF-α and the proportion of Th17 cells in the serum of CIA rats increased significantly, while the level of IL-10 increased slightly. This change was significantly reversed upon treatment with *L. casei* CCFM1074. Flow cytometry revealed that the proportion of Tregs among CD4^+^T cells in the mesenteric lymph nodes of CIA rats decreased, and the proportion of Th17 cells increased significantly. After treatment with *L. casei* CCFM1074, the opposite result was observed and the integrity of the intestinal tract was restored, which alleviated arthritic symptoms in CIA rats. These results indicated that *L. casei* CCFM1074 affects the skeletal system and slows the progression of RA by downregulating pro-inflammatory cytokines, rebalancing the Treg/Th17 cell ratio, and regulating intestinal microflora.

*Lactobacillus rhamnosus*, a probiotic strain, is a gram-positive anaerobic bacterium that transports and metabolises carbohydrates, thereby maintaining the integrity of the epithelial intestinal tract (177). Experimental research has found that *L. rhamnosus* significantly decreased the expression of the OC factors IL-6, IL-17, TNF-α, and RANKL in mice, and significantly increased the expression of anti-OC factors IL-4, IL-10, and IFN-γ, thus inhibiting OC proliferation and differentiation, and slowed bone loss. Furthermore, *in vivo* studies, revealed that *L. rhamnosus* significantly reduced the OVX mice percentage of OB CD4+Rorγt+Th17 cells from different immune sites, such as bone marrow, spleen, and lymphocytes, and significantly increased the percentage of CD4+Foxp3+Treg and CD8+Foxp3+Tregs in anti-OCs, which increased the percentage of Tregs, reduced the percentage of Th17 cells, regulated the balance of Th17/Treg cells, and inhibited bone resorption (178). This further demonstrates the immunomodulatory role of *L. rhamnosus* in regulating the balance of Th17/Treg cells, which opens a new avenue for the treatment of PMOP. In addition, some studies have found that the expressions of TNF-α, IL-17, RANKL, and RoR-γt were upregulated in the colon and bone marrow of OVX rats, while those of TGF-β, IL-10, and Foxp3 were downregulated. However, treatment with *L. rhamnosus* GG reversed these changes, downregulated the number of Th17 cells, and upregulated the number of Treg cells in the colon and bone marrow of rats, indicating that oestrogen deficiency damaged the intestinal barrier in OVX rats and increased intestinal permeability and Th17/Treg imbalance (179). The expression trends of Treg/Th17 cells in the intestines and bones were similar. These results suggest that *L. rhamnosus* GG improves OP induced by oestrogen deficiency by regulating the intestinal microbiome and intestinal barrier and stimulating the Th17/Treg balance in the intestine and bone. *Bifidobacterium longum*, a widely studied probiotic, is a gram-positive anaerobic bacterium that colonises the human gastrointestinal tract, regulates the diversity of microorganisms in the intestinal tract, and has immunomodulatory potential in relieving various inflammatory diseases (180). It was found that TNF-α, IL-6, and IL-17 increased, anti-OC factors (such as IFN-γ and IL-10) decreased, and OC factor levels increased in OVX mice. Further study showed that the intervention with *B. longum* significantly increased the percentage of Bregs and the production of IL-10 and IFN-γ, and decreased the production of TNF-α, IL-6, and IL-17. Bregs stimulated by *B. longum* significantly increased the percentage of Treg cells and IL-10 levels and significantly decreased the percentage of Th17 cells and IL-17 levels (181). These results suggested that Bregs stimulated by *B. longum* are powerful regulators of Th17-Treg cell differentiation.

Thus, *B. longum* can regulate the bone protective effect of the immune protein “Breg-Treg-Th17 cell axis”, thus opening up a new pathway for the treatment of inflammatory bone loss observed in PMOP.

### 4.3 Chinese medicinal compounds

Although probiotics have shown significant efficacy in treating RA and PMOP, and thus have developed into promising therapeutic agents (182), most Western medicines cause many adverse reactions and are expensive for long-term treatment (183). Therefore, it is important to identify alternative drugs to traditional medicines. Traditional Chinese medicine has the characteristics of multi-component, multi-channel, multi-target, and overall regulatory characteristics and are safe, have low toxicity and low cost, and have a long history in treating bone diseases, such as RA and PMOP (184, 185). Therefore, traditional Chinese medicine treatment can be used as an alternative therapy for long-term chronic diseases, such as RA and PMOP. Among these, traditional Chinese compounds are characterised by the monarch, minister, adjuvant, and envoy, principles, which emphasise comprehensive treatment based on multiple components rather than a single treatment (186, 187). Such multi-component treatments not only improves the curative effect and avoids serious side effects or drug resistance but also adjusts the Th17/Treg cell ratio and promotes the rebuilding of a new immune balance. Many studies have demonstrated the effectiveness of traditional Chinese compounds as supplementary or alternative therapies for RA and PMOP (188, 189). as summarised in **Table 4**.

**Table 4:** Therapeutic effects of Chinese medicine compound on RA and PMOP by regulating Th17/Treg balance.

Chinese herb prescription	Reduced immune inflammatory factors	Anti-inflammatory factors	Disease	Refs
Licorice and Aconite Decoction	miR-34a, Th17, ROR-γt, IL-17A, TNF-α, IL-1β, IL-6	Foxp3, Treg, IL-10	RA	205
Yunnan Baiyao	IL-17, Th17, RANKL, OPG	IL-10, Treg	RA	206
Soufeng sanjie formula	TNF-α, IL-6, IL-17A, ROR-γt, p-STAT3, IRF4, Th17	IL-10	RA	207
Erteng Tongbi Decoction	IL-6, TNF-α, IL-17, Th17	IL-10, Treg	RA	208
Xuebijing	IL-1β, IL-6, IL-17A, TNF-α, Th17	Treg	RA	209
Zishen Tongluo formula	IL-6, IL-23, IL-17, IL-21, TNF-α, GM-CSF, IFN-γ, ROR-γt, Th17	TGF-β, IL-2, IL-10, Treg, Foxp3	RA	210
Xianfanghuomingyin	IFN-γ, T-bet, IL-17, STAT3, ROR-γt, Th1, Th17	IL-4, GATA-3, Th2, Treg	RA	211
Qianghuo Erhuang Decoction	IL-6, IL-17, TNF-α, Th17	TGF-β, Treg	RA	212
Jiangu granule	CD4+ T, Th17, IL-6, IL-17, TNF-α, RANKL, Tracp-5b, CTX-1	Treg, IL-4, IL-10, TGF-β, OPG	PMOP	213
Bushen Huatan Recipe	IL-6, IL-6R, IL-17, ROR-γt, JAK2, p-JAK2, STAT3, p-STAT3, JAK1, p-JAK1, STAT5, p-STAT5, Th17	Foxp3, IL-10, TGF-β1, IL-2, Treg	PMOP	214
Guilu Bugu Prescription	Th17	Treg	PMOP	215
Zhuanggu Zhitong Capsule	ROR-γt, IL-17A	TGF-β, Foxp3, IL-10	PMOP	216
Jinkui Shenqiwan Combined with Buzhong Yiqitang	Th17, IL-17, TNF-α, RANKL, IFN-γ, Th17/Treg	Treg, IL-4	PMOP	217
Zuoguiwan	IL-17, IL-6, ROR-γt, Th17	TGF-β, BMD, Foxp3, Treg, Treg/Th17	PMOP	218-220

Traditional Chinese medicinal compounds have been reported to regulate the differentiation of Th17 or Treg cells through miRNAs and to participate in the occurrence and development of RA. For example, Zhao et al. (190) found that miR-34a is upregulated and Foxp3 is downregulated in CIA mice. After intervention with Gancao Fuzi Decoction, the gene expression of miR-34a gene expression was inhibited and Foxp3 protein expression was upregulated, which downregulated the proportion of Th17 cells in the spleen, the mRNA expression of ROR-γt and IL-17A, and the levels of pro-inflammatory factors, such as IL-17A, TNF-α, IL-1β, and IL-6 in the serum of CIA mice, and upregulated the proportion of Treg cells in the spleen of CIA mice, the mRNA expression of Foxp3 and IL-10, and the level of IL-10 in the serum. The pathological score of the CIA mice was significantly reduced, and joint swelling and bone injury improved. Gancao Fuzi Decoction regulates the Th17/Treg cell imbalance by targeting Foxp3 with miR-34a, thus playing an anti-RA role in CIA mice. Some studies have also found that Yunnan Baiyao treatment significantly reduced the level of IL-17 in the serum of CIA rats, significantly increased the level of IL-10, reduced the number of Th17 cells, and increased the number of Treg cells in the spleen of CIA rats, thereby reducing the ratio of Th17/Treg cells. In addition, Baiyao decreased the expression of RANKL and OPG in the joint tissues of CIA rats and inhibited RANKL-induced OC production (191). This indicates that Yunnan Baiyao exhibits anti-RA activity by regulating the balance between Th17 and Treg cytokines and inhibiting OC activation. In addition, Hua et al. (192)found that Soufeng Sanjie Recipe decreased the levels of inflammatory cytokines TNF-α, IL-6, and IL-17A in serum and joints of CIA mice, and increased the level of IL-10. Through *in vivo* and *in vitro* experiments, Soufeng Sanjie Recipe (SF) was further found to decrease the phosphorylation level of ROR-γt and STAT3 in spleens of CIA mice, inhibit the expression of interferon-regulated cytokine 4 (IRF4), reduce the number of Th17 cells and the production of IL-17 in the spleen and lymphocytes, and significantly inhibit the production of Th17 cells *in vitro*. The results indicated that SF inhibited the differentiation of Th17 cells by inhibiting the phosphorylation levels of ROR-γt, IRF4, IL-17A, and STAT3, and restored the Th17/Treg balance in the spleen and lymph nodes of CIA mice, which was very important for the treatment of RA. Other studies have found that Erteng Tongbi Decoction can significantly reduce the proportion of Th17 cells and increase the proportion of Treg cells in the spleen and lymphocytes by regulating T cell differentiation and cytokine balance, that is, inhibiting the production of IL-6, TNF-α, and IL-17, and promoting the expression of IL-10, thus repairing the balance of Th17/Treg cells and reversing the immune imbalance of CIA mice (193). The anti-RA effect of Erteng Tongbi Decoction may be directly related to the regulation of the cytokine balance. In addition, Clinical studies and animal experiments have confirmed that Xuebijing (XBJ) treatment decreases the levels of inflammatory cytokines IL-1β, IL-6, IL-17A, and TNF-α and the proportion of Th17 cells, and significantly increased the proportion of Treg cells in synovial fluid, spleen, and lymphoid tissue. XBJ may restore the balance of immune cells by increasing the number of Tregs and decreasing the proportion of Th17 cells, thus exerting a therapeutic effect in RA (194). A previous study also showed that Zishen Tongluo Recipe decreases the expression of inflammatory factors, such as IL-6, IL-23, IL-17, IL-21, TNF-α, GM-CSF, and IFN-γ, in the plasma of CIA mice, increases the levels of anti-inflammatory factors, such as TGF-β, IL-2, and IL-10, significantly decreased ROR-γt and Th17 cells, significantly upregulates Treg-related cytokines and Foxp3 mRNA and the ratio of Treg cells, restores the balance of Th17/Treg cells, and improves the symptoms of CIA mice (195). Nie et al. (196) also found through research that Xianfang Huomingyin significantly downregulates the abnormal differentiation of Th1 and Th17 cells and upregulates the differentiation of Th2 and Treg cells in the spleen and lymph of CIA mice by downregulating Th1-related cytokines, such as IFN-γ, T-bet, IL-17, STAT3, and ROR-γt, and upregulating Th2-related cytokines and transcription factors, such as IL-4 and GATA-3. By regulating the differentiation of Th1, Th2, and Th17 cells and promoting the differentiation of Tregs, the balance between T lymphocytes can be restored, thereby maintaining immune tolerance and reducing cartilage destruction and pannus formation. This balance also plays an important role in the treatment of RA. High-dose Qianghuo Erhuang Decoction significantly downregulated the expression of serum IL-6, IL-17, and TNF-α, upregulated the level of TGF-β, and improved synovial inflammation in adjuvant arthritis rats. In addition, high-dose Qianghuo Erhuang Decoction significantly increased the ratio of Treg cells in the spleen and decreased the ratio of Th17 cells (197). Moreover, a high dose of Qianghuo Erhuang Decoction was found to restore the balance between Th17 and Treg cells, inhibited arthritis and synovial hyperplasia, and reduce angiogenesis and articular cartilage destruction by regulating related cytokine networks, thereby exerting a therapeutic effect on RA.

Traditional Chinese medicine compounds not only play a role in treating RA by regulating the balance of Th17/Treg cells but are also involved in the treatment of PMOP by regulating the balance of Th17/Treg cells. For example, Sun et al. (189) found that Jiangu Granule (JGG) decreased the ratio of CD4^+^T and Th17 cells and the contents of IL-6, IL-17, and TNF-α secreted by Th17 cells in the spleen by reducing the permeability of the colon epithelium in OVX rats, while it also increased the ratio of Treg cells and the contents of IL-4, IL-10, and TGF-β secreted by Treg cells increased to varying degrees, thereby shifting the balance of Th17/Treg in favour of Tregs. Moreover, JGG has been shown to restore the Th17/Treg cell ratio by reducing intestinal epithelial permeability. In addition, the expression of OPG and RANKL, the key effective biomarkers of bone immune regulation, and the expression of Tracp-5b and CTX-1 were further detected. JGG increased OPG and decreased RANKL (the key effective biomarkers of bone immune regulation), Tracp-5b, and CTX-1 in the serum of OVX rats, and altered cytokines related to bone immune regulation. Therefore, JGG regulates the Th17/Treg balance through the intestinal microflora, thereby effectively preventing bone loss and enhancing bone strength. In addition, some studies have found that the concentrations of inflammatory factors IL-6, IL-17, and ROR-γt in OVX rat serum, the mRNA levels of IL-6, IL-6R, JAK2, STAT3, JAK1, STAT5, IL-17, and ROR-γt in femur tissue, and the protein levels of IL-6, IL-6R, JAK2, p-JAK2, STAT3, p-STAT3, JAK1, p-JAK1, STAT5, p-STAT5, IL-17, and ROR-γt were significantly increased by JGG treatment, while the concentrations of Foxp3, IL-10, TGF-β1, and IL-2, and the levels of Foxp3, IL-10, IL-2 mRNA, and protein in bone tissue decreased significantly. Flow cytometry revealed a significant decrease in the number of CD4+CD25+Foxp3 (Treg) cells, a significant increase in the number of CD4+IL-17A (Th17) cells, and a significant increase in the Th17/Treg ratio in the bone tissue of OVX rats. In addition, the concentrations of IL-6 and IL-17 in the colon tissues of OVX rats significantly increased, whereas those of IL-2 and IL-10 decreased. However, such alterations in OVX rats can be reversed by Bushen Huatan Recipe, indicating that the destruction of the intestinal barrier function leads to an intestinal immune response, which may lead to an immune response in the blood and bone tissue (198). Bushen Huatan Recipe may inhibit OC differentiation by reducing IL-6 levels and affecting Th17 cell expression through the IL-6/JAK2/STAT3 pathway. Increasing IL-2 levels and influencing Treg expression through the IL-2/JAK1/STAT5 signalling pathway promotes OB differentiation, further repairing the destruction of intestinal barrier function and inhibiting the immune response by adjusting the Th17/Treg balance, which plays a role in preventing and treating PMOP. Another clinical study found that Guilu Bugu Recipe regulates the expression of Th17, Tregs, and related factors; reverses the imbalance of Th17/Treg, inhibits the expression of pro-inflammatory factors; improves the expression of bone mineral density and oestrogen; and reduces bone loss, thus playing a role in reducing PMOP (199). In OVX rats, Zhuanggu Zhitong recipe increased the concentration OVX rats of TGF-β in the spleen lymphocytes and the expression of Foxp3 and IL-10, and the percentage of Foxp3 cells in bone tissue of in a dose-dependent manner, decreased the expression of ROR-γt and IL-17A, and the percentage of ROR-γt cells in spleen lymphocytes and bone tissue, and increased the ratio of Th17/Treg cells, thus regulating the balance of Th17/Treg in OVX rats and maintaining the balance of bone metabolism (200). In a clinical study, An et al. (201) also identified T cell subsets in patients with PMOP, revealed the imbalance of Th17, Treg cells, and related cytokines, and showed that Th17/Treg imbalance leads to an increase in IL-17, the effector factor secreted by Th17. The increase of IL-17 further induces local inflammatory reactions, and the production of RANKL and TNF-α stimulates the production of OCs. In addition, inflammatory factors, such as TNF-α and IL-17, can further aggravate inflammatory reaction. The levels of Treg and IL-4 in the observation group were higher than those in the control group, while cytokines, such as Th17, IL-17, TNF-α, and IFN-γ, and the Th17/Treg ratio were lower than those in the control group, suggesting that the oral administration of Jinkui Shenqi Pill combined with Buzhong Yiqi Decoction regulates T cell subsets, promotes Th17/Treg to return to normal, inhibits the expression of pro-inflammatory factors and OC production, and is beneficial for increasing bone mass and the treatment of PMOP. In addition, many studies in OVX mice found that the proportion of Th17 cells increased significantly, the proportion of Treg cells decreased significantly, and the balance of Th17/Treg cells was shifted towards Th17 cells (202–204). However, Zuo Gui Wan reduced the mRNA and protein expression levels of IL-17, IL-6, ROR-γt, and the proportion of Th17 cells in OVX mice in a dose-dependent manner, and it also increased the mRNA and protein expression levels of TGF-β, BMD, Foxp3, and the proportion of Treg cells, which shifted the balance of the Th17/Treg ratio towards Tregs, thus inhibiting the production of osteolytic cytokines and reducing bone loss. This indicates that regulating the Th17/Treg balance is an effective mechanism for alleviating bone loss induced by oestrogen deficiency and provides a new method for the clinical treatment of PMOP.

### 4.4 Monomers of traditional Chinese medicine

With the integration of traditional and modern medicine, an increasing number of studies have found that traditional Chinese monomers have the dual advantages of traditional Chinese medicine and chemical medicine (205), which can optimise their functions. Therefore, to maximise the curative effect, adequacy of theory, and achieve better integration of traditional Chinese and Western medicines, we will pay more attention to the treatments of diseases using traditional Chinese medicine monomers should be further investigated (206). Recent studies have found that traditional Chinese medicine monomers have a regulatory effect on immune-bone remodelling and play a therapeutic role in RA and PMOP by regulating the Th17/Treg cell balance (207). The results are summarised in **Table 5**.

**Table 5:** Therapeutic effects of Chinese medicine herbal monomers on RA and PMOP by modulating Th17/Treg balance.

Chinese herbal monomer	Reduced immune inflammatory factors	Anti-inflammatory factors	Disease	Refs
evodiamine	TNF-α, IL-1β, IL-6, IL-17, p-STAT3, Th17	IL-10, p-STAT5, Treg	RA	224
curcumin	Th17, IL-17	Treg, TGF-β	RA	225
Cryptotanshinone	IL6, IL-17, p300, p-STAT3, ROR-γt, Th17	Foxp3, TGF-β1, IL-10	RA	226
quercetin	IL-17A, IL-21, ROR-γt TNF-α, IL-1β, IL-6, PGE2, COX-2, NLRP3, Caspase-1, Th17	IL-10, TGF-β, Foxp3, Treg	RA	227-228
oxymatrine	TNF-α, IL-17A, ROR-γt, Th17	Foxp3, Treg	RA	229
Diosgenin	Th17, IL-17, ROR-γt	Does not affect expression of IFN-γ, Foxp3, IL-10, IL-6, IFN-γ	RA	230
Leonurine	TAZ, IL-17, IL-6, IL-1β, TNF-α, Th17	IL-10, Treg	RA	231
Cinnamtannin D1	AHR, IL-17, IL-6, IL-1β, ROR-γt, Th17, P-STAT3/ROR-γt	IL-10, TGF-β, Foxp3, p-STAT5/Foxp3, Treg	RA	232
Naringenin	RANKL, CTSK, c-Fos, DC-STAMP, NFATc1, TRAP, V-ATPase d2, F-actin, IL-1β, IL-17, Th17	Treg, IL-4, Th2, Treg	PMOP	233
psoralen	IL-17, TNF-α	IL-10, TGF-β	PMOP	234
Baicalin	p-STAT3, Th17, IL-17A, IFN-β, TNF-α, IL-1β, IL-23, IL-27, Th17/Treg	/	PMOP	235

For example, the expression of TNF-α, IL-1β, and IL-6 in the serum and synovium of AA rats was basically restored to the control level after evodiamine intervention. Further studies showed that evodiamine decreased IL-17 and p-STAT3 levels in the spleen and increased IL-10 and p-STAT5 levels. It is well known that IL-17 and p-STAT3 are known to promote the differentiation of Th17 cells, and IL-10 and p-STAT5 can promote the differentiation of Treg cells. Therefore, evodiamine treatment regulates the abnormal expression of Tregs and Th17 cells in the spleen, enhances the proliferation of Tregs, inhibits the proliferation of Th17 cells, and regulates the balance between Th17/Treg cells. This further confirmed that the antirheumatic effect of evodiamine may be related to its inhibition of synovitis and regulation of the Th17/Treg balance (208). In addition, Liu et al. (209) performed clinical research and found that after curcumin intervention in patients with RA, the percentage of Th17 cells and IL-17 levels decreased significantly, while that of Treg cells and TGF-β levels increased significantly. The results indicated that curcumin could specifically reduce the differentiation of Th17 cells in CD4+T cells of patients with RA *in vitro* and promote their differentiation into Treg cells, and regulate the function of Th17 and Treg cells and the balance of Th17/Treg cells by reducing IL-17 and increasing TGF-β. This ability to regulate the Th17/Treg cell balance specifically affects CD4^+^ T cells in patients with RA, but not in healthy individuals. Thus, curcumin may be a novel drug for the treatment of RA. Cryptotanshinone has been shown to increased the expression of Foxp3, TGF-β1, and IL-10 related signal molecules to induce Treg cell differentiation in CIA mice in a dose-dependent manner, and it significantly decreased the concentration and mRNA level of IL-6 and IL-17 in the serum and joints. Further experiments showed that cryptotanshinone downregulates p300 expression, inhibits p300-mediated p-STAT3, and inhibits the mRNA level of Th17 cells and ROR-γt, a key transcription factor in the differentiation of Th17 cells, and regulates Th17/Treg imbalance (210), Thus, cryptotanshinone represents a potential immunomodulator for RA therapy. In CIA rats, quercetin administration has been shown to decrease the contents of Th17-related cytokines IL-17A, IL-21, and ROR-γt and inflammatory mediators TNF-α, IL-1β, IL-6, PGE2, and COX-2 which play a key role in the development of RA, decreased the protein expression levels of inflammatory corpuscles NLRP3, Caspase-1, and IL-1β, and increased the expression of Treg-related cytokines IL-10, TGF-β, and Foxp3. The percentage of Th17 cells decreased, which inhibited inflammatory reactions and OC production and restored the Th17/Treg balance (211, 212). This indicated that quercetin inhibits the activation of inflammatory corpuscles, such as NLRP3 and differentiation of OCs by regulating the balance of Th17/Treg cells and alleviating the manifestation of RA. Oxymatrine reduces inflammatory cytokines, such as TNF-α and IL-17A, in the spleen and serum of CIA rats in a dose-dependent manner, downregulates the mRNA and protein levels of ROR-γt related to Th17 cells, and upregulates the mRNA and protein levels of Foxp3 related to Treg cells, which significantly reduced the severity of disease in CIA rats and eliminated symptoms, such as claw swelling, arthritis score, and synovial hyperplasia. Because the downregulation of IL-17 and Th17 cells and upregulation of Treg cells are important factors in inhibiting inflammation, oxymatrine can play an anti-inflammatory role in autoimmune arthritis by regulating the Th17/Treg imbalance and can be used as an immunosuppressive and cartilage-protective drug (213). *In vivo* and *in vitro* experiments showed that dioscin treatment significantly reduced Th17 cell differentiation, inhibited IL-17 production, and downregulated the mRNA expression of IL-17 and ROR-γt mRNA expression in CIA mice; however, it failed to change the ratio of IFN-γ to Foxp3 and the mRNA expression of IL-10, IL-6, and IFN-γ in CD4^+^ T lymphocytes (214). These results indicated that dioscin improved the symptoms of CIA in mice by inhibiting Th17 cell differentiation without affecting the differentiation of Th1 and Treg cells, thus providing an experimental basis for further studies on the clinical application of dioscin in treating RA. Traditional Chinese medicine monomers not only inhibit the expression of Th17 cells and their related secretory factors, but also promote the expression of Treg cells and their related secretory factors, regulate the balance of Th17/Treg cells, inhibit pathogenic cytokines, and restore the balance of Th17/Treg cells to treat RA. For example, recent experimental studies have indicated that the transcription regulator TAZ, a molecule involved in Th17 development and the imbalance between Treg and Th17 cells, induces a Th17/Treg imbalance in patients with RA patients by promoting Th17 cell differentiation and inhibiting Treg cell differentiation. Leonurine can inhibit the expression of TAZ, reduce the levels of inflammatory factors IL-17, IL-6, IL-1β, and TNF-α, increase the expression of anti-inflammatory cytokine IL-10, increase the proportion of Treg cells, decrease the proportion of Th17 cells, reverse the Treg/Th17 imbalance induced by TAZ, and alleviate arthritis inflammation (215). In addition, Shi et al. (216) found through experimental research that cinnamon tannin D1 downregulated inflammatory cytokines IL-17, IL-6, and IL-1β in the serum of CIA mice and upregulated IL-10 and TGF-β. ROR-γt and IL-17 mRNA levels in Th17 cells were downregulated, and Foxp3, IL-10, and TGF-β mRNA in Tregs were upregulated. The percentage of Th17 cells decreased, while that of Treg cells increased. These results indicate that cinnam on Tannin D1 inhibited the differentiation of Th17 cells, promoted the differentiation of Treg cells, and restored the balance of Th17/Treg cells in CIA mice. *In vitro* experiments further found that this effect of cinnamon tannin D1 may be related to downregulating P-STAT3/ROR-γt to inhibit Th17 cell differentiation and upregulating p-STAT5/Foxp3 to promote Treg differentiation, indicating that cinnamon tannin D1 regulates the Th17/Treg balance to inhibit excessive immune response. Aryl hydrocarbon receptor (AHR) is a ligand-activated transcription factors, and recent evidence has shown that AHR is an important regulator of the differentiation between Th17 and Tregs. When AHR was knocked down, the balanced regulation of cinnamon tannin D1 on Th17 and Treg cells was eliminated; however, this effect was impaired when AHR was overexpressed, this effect was impaired. These results indicated that cinnamon Tannin D1 regulates the balance of Th17/Treg cells by inhibiting the production of AHR and alleviating the symptoms of CIA.

Traditional Chinese medicine monomers can regulate the balance of Th17/Treg cells to treat RA and have the same effect on PMOP. Experimental studies have found that naringenin significantly decreases the expression of OC-related factors, such as cathepsin K(TSK), c-Fos, DC-STAMP, NFATc1, TRAP, and V-ATPase d2, at the mRNA and protein levels in a concentration-dependent manner, thereby significantly reducing bone resorption. In addition, further *in vitro* studies revealed that after naringenin treatment of T cells, the size and number of F-actin rings decreased significantly, and the expression levels of IL-1β and IL-17 decreased, which inhibited the proliferation and activation of Th17 cells and significantly reduced the percentage of Th17 cells. The expression of IL-4 and the percentages of Th2 and Treg cells were significantly increased by Treg cell induction. Although anti-IL-4 antibody reversed the effects of naringenin, anti-RANKL blocked the effects of anti-IL-4, indicating that naringenin regulates Th17/Treg cells by promoting the release of IL-4 from T cells, and inhibiting the expression of OC-specific markers induced by RANKL; thus, it plays an important role in the prevention and treatment of PMOP (217). *In vivo* experiments showed that psoralen significantly increased serum and bone levels of IL-10 and TGF-β in OVX rats, but decreased the levels of IL-17 and TNF-α. IL-10 and TGF-β are mainly produced by Tregs in CD4+T cells, while IL-17 and TNF-α are mainly produced by Th17 in CD4+T cells. Tregs and Th17 cells are two subsets of T cells with opposite functions in CD4+T cells. Psoralen may play an anti-PMOP role by regulating the functional balance between Tregs and Th17 cells among CD4^+^ T cells (218). In addition, Chen et al. (219) performed experimental studies and found that high doses of neobaicalein inhibited the differentiation of Th17 cells and the secretion of related cytokines, such as IL-17A, IFN-β, and TNF-α, during Th17 cell differentiation, reduced the expression of IL-1β, IL-23, and IL-27, and significantly downregulated the ratio of Th17/Treg cells, Therefore, neobaicalein is expected to play a role in treating PMOP by regulating the Th17/Treg ratio.

### 4.5 Other treatments

#### 4.5.1 A new target for targeting and regulating Th17/Treg balance therapy of RA and PMOP

With the advancement of medical research, new targets have been reported that regulate the balance of Th17/Treg cells (**Table 6**). which is important for the treatment of immune bone diseases (220). Among these, lipase D (PLD) is considered a promising target for the treatment of inflammation and plays a vital role in various inflammatory and autoimmune diseases (221). Studies have shown that PLD expression is positively correlated with RA severity. Therefore, using PLD knockout mice and selective Lipase D inhibitors, we found that the use of selective PLD inhibitors could alleviate pathological bone destruction in CIA mice by inhibiting OC production and bone resorption. In addition, selective inhibitors of lipase D increase the differentiation of Tregs and inhibit the differentiation of Th17 cells. These results indicated that PLD could promote RA by targeting Th17 and Treg cells, which unbalanced the Th17/Treg ratio, promoted OC proliferation, and promoted RA (222). In addition, another study found that maresin 1 (MaR1), a newly discovered mediator produced by docosahexaenoic acid (DHA) in macrophages, can inhibit inflammation and regulate the immune response (223). An experimental research study showed that, Mar1 decreases the levels of Th17 cell-related factors IL-17, TNF-α, IFN-γ, IL-1β, and IL-6 and the expression of Th17 transcription factor RORc by upregulating mir-21, and increased the levels of Treg cell-related factors IL-10 and TGF-β and the expression of Treg transcription factor Foxp3 (224). This indicates that Mar1 is a therapeutic target for RA and can effectively improve the progression of RA by regulating the Th17/Treg imbalance. IL-2-inducible T-cell kinase (ITK) plays an important role in the differentiation of T helper subsets, and its inhibitory effect has been recognised as a treatment for T cell-mediated inflammatory diseases. ITK kinase levels are significantly increased in CD4 + T cells of patients with RA, animal experiments, it was further revealed that an ITK inhibitor downregulated Th17 cells and effectively upregulated Treg cells by regulating Foxo1 translocation, which significantly inhibited the transformation of Treg cells into Th17 cells and restored the balance of Th17/Treg cells by downregulating the PI3K-Akt-mTOR signalling pathway, indicating that blocking ITK may be an effective strategy to treat RA (225). DJ-1 consists of 189 amino acids and plays an important role in T cell differentiation. DJ-1 has been shown to inhibit the differentiation of pathological Th cell subsets (Th17, RANKL+CD4 + T cells) and the production of pro-inflammatory cytokines IL-17A and TNF-α, induce the differentiation of Tregs, weaken the expression levels of OC-related factors TRAP, ATP6v0d2, NFATc1, and CTSK, inhibit the production of OCs induced by RANKL and IL-17A, and regulate the balance of Th17/Treg, thereby playing an important therapeutic potential in the pathogenesis of RA (226). TAGAP is a RhoA-specific GTPase activator that induces the differentiation of human CD4 + T cells into T cells, and the expression of TAGAP, RhoA and NLRP3 are significantly increased in patients with RA RA and CIA rats. Inhibiting the expression of TAGAP significantly decreased the protein content of RhoA and NLRP3 in CD4+T cells, the relative expression levels of serum inflammatory factors TNF-α, IL-1β, and IL-17, and the relative mRNA expression levels of pro-inflammatory cytokines (TNF-α, IL-6, and IL-1β), MMP-3, and MMP-13, whereas it significantly increased the expression of anti-inflammatory cytokines IL-10 Moreover, reducing TAGAP expression promoted the differentiation of Th17 cells, inhibited the differentiation of Treg cells *in vitro* and *in vivo*, and restored the balance between Th17 and Treg cells. The results showed that inhibiting TAGAP inhibited the expression of NLRP3 and RhoA, which eventually led to a decrease in Th17 cell differentiation and an increase in Treg cell differentiation, and finally improved the severity of RA, thus providing a new experimental foundation for targeting TAGAP as a therapeutic agent for RA (227). Protective protein DX (PDX) is a protective protein D1 isomer that belongs to the special decomposition promoting medium (SPM) family and is derived from ω 3 long chain polyunsaturated fatty acid (ω 3 LC-PUFA), Moreover, serum PDX is a potential biomarker of RA activity (228). Clinical studies have shown that PDX levels in patients with RA decrease during the active period and increase during the inactive period. XX constructed a CIA mouse model, and found that PDX obviously delayed RA progression in CIA, upregulated the mRNA level of Tregs cells and Treg characteristic transcription factor FOXP3 and the expression of anti-inflammatory cytokines IL10 and TGF-β, but downregulated the mRNA level of Th17 cells, Th17 characteristic transcription factor ROR-γ t and the expression of pro-inflammatory cytokines IL-1β, IL-18, IL-6, TNF-α, and IL-17A. In addition, further studies showed that PDX decreased the mRNA and protein levels of NLRP3 and the levels of IL-1β and CASP-1 related proteins of NLRP3 *in vitro*, while the overexpression of miR-20a also decreased the expression of NLRP3, CASP-1, and IL-1β, which was particularly obvious under the intervention of PDX. PDX also reduced the expression of NLRP3 through miRNA-20a and restored the balance of Treg/Th17 cells, which effectively improved the progression of CIA. PDX has been shown to inhibit NLRP3 inflammatory corpuscles through miR-20a to restore the Th17/Treg cell balance and effectively improve RA (229). At present, there are few studies on the treatment of PMOP by regulating the balance of Th17/Treg cells mediated by new targets. However, such studies on RA insights for the prevention and treatment of PMOP and the research and development of new drugs, thus continuous research and exploration are warranted.

**Table 6:** Novel targets for targeted modulation of Th17/Treg balance in the treatment of RA and PMOP.

Target	Mechanism of action		Treat enlightenment	Diseases	Refs
	Downregulation	Up-regulation			
PLD	PLD inhibitors: Th17	Treg	Targeted inhibition of PLD	RA	238
MaR1	IL-17, TNF-α, IFN-γ, IL-1β, IL-6 and RORc	mir-21, IL-10, TGF-β, Foxp3	Targeted activation of MaR1	RA	240
ITK	ITK inhibitors: Th17, PI3K-Akt-mTOR signaling pathway	Treg	Targeted inhibition of ITK	RA	241
DJ-1	Th17, IL-17A, TNF-α, TRAP, ATP6v0d2, NFATc1 and CTSK	Treg	Targeted activation of DJ-1	RA	242
TAGAP	TAGAP inhibitors: NLRP3, TNFα, IL-1β, IL-17, IL-6, MMP-3 and MMP-13	IL-10	Targeted inhibition of TAGAP	RA	243
PDX	Th17, ROR-γt, IL-1β, IL-18, IL-6, TNF-α, IL-17A, NLRP3, IL-1β Wa CASP-1	Tregs, FOXP3, IL10, TGF-β and miR-20a	Targeted activation of PDX	RA	245

#### 4.5.2 New drugs and methods for targeted recovery of Th17/Treg balance in the treatment of RA and PMOP

Th17/Treg imbalance is the main cause of RA and PMOP, therefore, identifying drugs to regulate the Th17/Treg balance is key to treating RA and PMOP. See **Table 7**. Li et al. (230)found that arsenic trioxide (As2O3) significantly reduced the expression of inflammatory cytokines, such as IL-17A, MMP13, IL-23, IL-6, STAT3, and ROR-γt, and enhanced the expression of IL-10, Foxp3, TGF-β1, and STAT5 by inhibiting the expression of STAT3. In addition, As2O3 decreased the percentage of Th17 cells, increased the proportion of Tregs in a dose-dependent manner, and enhanced the differentiation of CD4 + T cells into Tregs. These results suggest that As2O3 may be a potential immunomodulator for the treatment of RA by regulating the Treg/Th17 cell balance, improving joint destruction, and alleviating inflammatory reactions and pathological manifestations. Moreover, clinical studies have found that the absolute count and proportion of Treg cells decreased significantly in all patients with RA with DAS 28 scores ≤ 3.2, while the difference in the number of Th17 cells was not significant, indicating that the decrease of Treg cells may be the main reason for the imbalance of Th17/Treg in patients with RA with DAS 28 scores ≤ 3.2. However, the administration of rapamycin (common name sirolimus) increased the number of circulating Treg cells and significantly decreased the ratio of Th17/Treg cells, which indicated that sirolimus could effectively expand Treg cells in patients with RA with DAS 28 scores ≤ 3.2, thus restoring the healthy balance of Th17/Treg cells, which may improve the possibility of long-term and sustained clinical remission, reduce the probability of RA disease onset and change the clinical practice of routine treatment of RA (231). Sinomenine (SIN) is an isoquinoline alkaloid with biological activity. Studies have shown that SIN regulates the intestinal microflora by significantly enriching paracasein and Lactobacillus casei, regulating tryptophan metabolism, activating the SIN ligand AhR, improving microbial imbalance and intestinal barrier dysfunction, significantly reducing the mRNA levels of IL-17 and RORct in the synovium and colon of CIA rats, and increasing the expression of AhR, CYP1A1, Foxp3, and IL-10. Downregulation of Th17 cells and upregulation of Treg cells corrected the imbalance of Th17/Treg in the synovial fluid and colon. In addition, SIN plays an anti-RA role by regulating the differentiation of Th17 and Treg cells in the intestinal tract and enhancing their migration of Th17 and Treg to the inflammatory joints and synovium (232). These drugs provide a promising methods for targeting key metabolites or bacteria in the treatment of RA. In addition, another study found that Duanteng Yimu Decoction (DTYMT) improved the joint injury of CIA mice, inhibited the expression of ROR-γt, increased the expression of Foxp3, significantly downregulated the levels of IL-1β, IL-17, and TNF-α mRNA in primary T, and increased the level of IL-10 mRNA. In addition, drug intervention with DTYMT inhibited the differentiation of Th17 cells and promoted the production of Tregs in CIA mice, thus restoring the Treg/Th17 balance and inhibiting the proliferation, migration, and invasion of RA fibroblast-like synovial cells (233). These results suggest that DTYMT exerts anti-inflammatory activity in RA by regulating the release of inflammatory factors and the balance of Th17/Treg cells.

**Table 7:** New drugs targeting modulation of Th17/Treg balance for the treatment of RA with PMOP.

Medicines	Downregulation	Up-regulation	Disease	Refs
arsenic trioxide (As2O3)	IL-17A, MMP13, IL-23, IL-6, STAT3, ROR-γt, Th17	IL-10, Foxp3, TGF-β1, STAT5, Treg	RA	246
Rapamycin	Th17, Th17/Treg	Treg	RA	247
Sinomenine (SIN)	IL-17, RORct, Th17	AhR, CYP1A1, Foxp3, IL-10, Treg	RA	248
Duan Teng Yi Mu Tang (DTYMT)	ROR-γt, IL-1β, IL-17, TNF-α, Th17	Foxp3, IL-10, Treg	RA	249

In addition to developing and exploring new drugs that target the regulation of the Th17/Treg balance to treat RA and PMOP, it is also very important to study new therapeutic methods and administration routes. See **Table 8**. With the continuous research on traditional Chinese medicine, such treatments have gradually become recognized internationally, among which acupoint therapy is considered a popular adjuvant therapy for RA and has been recognised by researchers worldwide (234). Some studies have found that moxibustion with Zusanli and Shenshu can effectively inhibit arthritis in CIA mice, increase the number of Tregs, and decrease the number of Th17 cells (235). Therefore, reversing the Th17/Treg cell imbalance is related to the pathogenesis of RA. Studies have found that acupuncture builds a bridge between drugs, providing insights into the combination of traditional and modern medicine. For example, acupoint nanocomposite hydrogel-simulated acupuncture and moxibustion to target triptolide inhibited pro-inflammatory cytokines TNF-α, IL-6, IL-1β, IL17A and Th17 cells, but upregulate anti-inflammatory cytokines TGF-β and IL-10, and Treg cells, and reduced synovitis and cartilage erosion. These results indicate that triptolide may have a therapeutic effect on RA by promoting the differentiation of Tregs and inhibiting the differentiation of Th17 cells by simultaneously targeting a variety of cytokines and reconstructing the immune balance of Th17/Treg cells. Compared to triptolide alone, acupoint nanocomposite hydrogel simulated acupuncture that targets triptolide administration effectively reduced the toxicity and side effects of triptolide, explored the potential of acupoint administration, and provided ideas for developing a new drug delivery system with practical targeting abilities (236). In reports (237), Chinese medicine application technology has been recognised by patients because of its advantages in reducing toxic damage to the gastrointestinal tract, liver, kidney, and other organs, its convenient use, and its therapeutic effects in RA. For example, Yao et al. (238) performed animal experiments and showed that the external application of Wuteng ointment greatly inhibited the expression levels of inflammatory cytokines IL-17, TNF-α, IL-1, IL-6, and RNAKL, and improved the expression of anti-inflammatory cytokines IL-10 and TGF. Through further experiments, it was found that different doses of Wuteng ointment significantly reduced the ROR-γt level in spleen tissue, increased the Foxp3 level, altered the Th17/Treg cell ratio, improved the Th17/Treg cell imbalance in CIA rats, inhibited bone destruction, and played an effective therapeutic role in RA. According to the current research situation at home and abroad, there are many new drugs, new targets, and new methods that regulate the balance of Th17/Treg cells in RA; however, the research on PMOP is slightly insufficient. However, both RA and PMOP are bone immune diseases, and the results obtained for RA are believed to have certain value and research implications for PMOP. Thus, these connections warrant further study.

**Table 8:** Novel approach to target modulation of Th17/Treg balance for the treatment of RA with PMOP.

Category	Method	Downregulation	Up-regulation	Disease	Refs
moxibustion therapy	Moxibustion treatment for Zusanli and Shenshu	Th17	Treg	RA	251
acupuncture therapy	Acupoint nanocomposite hydrogels mimic acupuncture for targeted drug delivery of Triptolide	TNF-α, IL-6, IL-1β, IL17A, Th17	Treg	RA	252
Traditional Chinese medicine patch therapy	External application of Wuteng ointment	IL-17, TNF-α, IL-1, IL-6, RNAKL, ROR-γt, Th17/Treg	IL-10, TGF-β, Foxp3	RA	254

Based on the above research, we can conclude that both Western medicine and traditional Chinese medicine. However, these two treatment methods have achieved a certain clinical efficacy, although differences were observed between them. Conventional synthetic DMARDs, Namely, bDMARDs and tsDMARDs, are the most commonly used drugs in Western medicine for the treatment of RA (239). Although conventional synthetic DMARD have a strong anti-inflammatory effect, their effect is slow, and they are usually administered used for several weeks or even months, and its produces adverse gastrointestinal reactions and liver and kidney toxicity, thereby limiting its clinical application (240). Although bDMARD have a rapid and strong anti inflammatory effect in the treatment of RA, they can prevent bone destruction. And quickly relieves the disease symptoms. However, such drugs are expensive and causes serious infection-related complications. Compared with other drugs, patients have poor tolerance and compliance with bDMARDs, and a small number of patients develop drug resistance (241).

tsDMARDs can block inflammatory progression at the source, have a strong anti-inflammatory effect, are convenient to take orally, have similar effectiveness and safety as biological agents, and are slightly lower in price. However, diarrhoea, elevated serum total cholesterol and lipoprotein levels, pulmonary embolism, and other adverse reactions may occur during use (242). In the process of clinical application, it was found that, according to the clinical symptoms of patients and different activity stages of RA, two different antirheumatic drugs or antirheumaticdrugs could be combined with nonsteroidal anti-inflammatory drugs and glucocorticoids according to the clinical symptoms of patients and different activity stages of RA. This combined scheme not only achieves therapeutic effects but also increases treatment costs and adverse reactions, besides, Thus, owing to the long treatment cycle and high price of RA treatments and the large number of patients with RA who show poor drug compliance, the treatment effect is reduced and medical resources are wasted (243).

Commonly used Western medicines for the treatment of PMOP are bone resorption inhibitors and bone formation enhancers. Among them, bone resorption inhibitors can inhibit bone resorption through different mechanisms, although their long-term use has side effects, such as gastrointestinal discomfort, cardiovascular events, thromboembolism, cancer, and renal function damage. Bone formation promoters not only promote bone formation but also have side effects, such as nausea, dizziness, headache, palpitation, and increased risk of osteosarcoma (244, 245). Moreover, the medication treatment cycle for PMOP is at least one year, During this treatment process, because of the many side effects, high costs, and large individualised differences, patient compliance is poor. Thus the expected clinical treatment effect has not been achieved (246).

Traditional Chinese medicine is characterised by syndrome differentiation, disease combination, and the overall regulation, prevention, and treatment of RA and PMOP. By giving full play to its multi-target, multi-component, and multi-channel advantages, it can achieve the same effect as Western medicine; at the same time, it has few side effects, low price, convenient oral administration, high recognition and acceptance of patients, and has great potential in the treatment of RA and PMOP (247, 248). In addition, traditional Chinese medicine compounds can not only improve the curative effect but also avoid serious side effects or drug resistance through multi-component comprehensive treatment (249). Traditional Chinese medicine monomers not only have the dual advantages of traditional Chinese medicine and chemical medicine but also have a variety of medicinal characteristics. Moreover, they represent an important source of new drug research, development, and preparation, and plays an important role in innovative drug development (250). Moreover, in cases where using traditional Chinese medicine or Western medicine alone is not ideal in the clinical treatment process, the combination of traditional Chinese and Western medicine can be selected according to the clinicopathological characteristics and types, therefore, the quality of life of patients with RA and PMOP can be effectively improved by exploiting the strengths of each type of medicine (251, 252).

## 5 Quality of life and nursing care of patients with RA and PMOP

RA, as a chronic autoimmune disease with no lifelong cure, has a significant and varied impact on patients’ health-related quality of life (HRQoL), mainly in the areas of physical and mental health (253). A previous survey showed that more than three-quarters of patients with RA will experience chronic pain within 5 years after diagnosis, and long-term pain will lead to the deterioration of clinical symptoms of RA patients, which will reduce their physical and mental health and increase their risk of depression (254). Recent studies have confirmed that depression and anxiety increase the risk of RA, in addition, depression and anxiety can lead to panic and a lack of self-confidence in patients with RA, which is closely related to an increase in mortality (255). Other studies have found that the occurrence of RA is closely related to individual lifestyles, and smoking is recognised as a risk factor for RA and depression (256). Further, patients with RA are worried that exercise aggravates joint inflammation and pain and accelerates joint injury; therefore, exercise is limited. Studies have confirmed that routine exercise in patients with RA has the benefits of relieving pain, improving muscle function, and delaying disability attacks and does not have harmful effects on joints (9). In addition to the factors that induce the occurrence and development of RA and associated declines in the quality of life of patients with RA, a series of complications caused by RA also seriously reduce the quality of life of patients with RA, such as fatigue, corneal diseases, and sleep disorders. Fatigue is a common and serious complication that may arise from disease activity, chronic pain, sleep disorders, poor mood, and other reasons, and it has a great impact on patients’ lives, fatigue is classified into physical, life, cognitive, and emotional fatigue, In patients with RA, total fatigue>physical fatigue>life fatigue>cognitive fatigue>emotional fatigue, moreover total fatigue is positively correlated with depression, anxiety, disability, and helplessness (257). Other studies have found that patients with RA are complicated by a series of corneal diseases, including mild symptoms to severe corneal ulcers and corneal melting with visual loss, which will lead to a serious decline in the quality of life of RA patients (258). In addition, sleep disorders are a common clinical symptom of patients with RA, including non-restorative sleep, repeated waking at night, insomnia, and lethargy, which in turn leads to fatigue and daytime lethargy and subsequently to mental and physical fatigue, emotional disorder, daytime lethargy, and poor quality of life (259). In addition to the complications of RA, that seriously reduce the quality of life of patients with RA, this study also found that patients with RA have low work efficiency owing to joint inflammation and stiffness, and their income is reduced because they are forced to leave or change jobs. However, the treatment drugs for RA are expensive, which further increases the economic burden on families and reduces the overall quality of life of patients with RA (260). The survey also found that patients with RA in poor rural areas were worse off and had a lower quality of life than patients with RA in cities (261).

Considering the present situation of RA, it is necessary to strengthen RA nursing, that is, to strengthen education and vaccinations for preclinical RA, such as in people who do not meet RA standards, improve people’s understanding of RA through education and publicity, consciously promote exercise and lifestyle changes to avoid RA, and prevent RA through vaccination (262). Moreover, the state should pay more attention to RA, expand the scope of medical insurance, and reduce the economic burden of medical treatment for patients with RA, at the policy level, while medical staff should improve their working abilities and enhance their knowledge, strategies, and skills to communicate with specific patient groups to strengthen the psychological counselling of patients with RA, reduce the physical and mental pressure on patients with RA, and enhance the confidence in treatment. At the same time, to improve patient compliance, it is necessary to redistribute privileges, avoid the preferred methods of paternalistic medicine, and increase patients’ rights to participate in decision-making, Moreover, the monitoring of disease activity, screening for potential tuberculosis infections, and screening for complications before the use of biological agents must be improved (263, 264). In addition, in poverty-stricken areas, contact with medical staff via telemedicine technology can provide guidance, improve the medical level of rural health services, and narrow the medical gap (265).

PMOP is a silent bone disorder, and common symptoms that gradually develop as the disease progresses include back pain, osteoporotic fractures, spinal deformities, and multiple organ dysfunction gradually develop, among the complications of PMOP, osteoporotic fractures are the most common, including hip, wrist, and vertebral fractures (266). Hip fractures are the most significant manifestation of this disease in terms of morbidity, mortality, and medical expenses. In addition, the problems of physical pain, limited physical function, long recovery cycle, numerous complications, and high treatment costs after hip fracture surgery have caused a huge economic burden and mental pressure on society and families, thereby seriously reducing the quality of life of patients with PMOP (267). Health-related quality of life (HRQoL) is a comprehensive evaluation index of physical, emotional, and social health, and a study found that although the total HRQoL of PMOP patients was poor, but the HRQoL of postmenopausal osteoporotic fracture women was worse than that of postmenopausal osteoporotic women, indicating that PMOP fracture is the main reason for reduced HRQoL in women (268). Another study found that the bone mineral density of the femoral neck and lumbar vertebrae of PMOP women with PMOP was positively correlated with HRQoL, whereas the degree of brittle fracture was negatively correlated with HRQoL, at the same time. Moreover, the prognosis of fractures in turn affects HRQoL, which is worse after fracture recovery than before the fracture, indicating that more research is needed to prevent PMOP (269).

The incidence of osteoporosis and osteoporotic fractures can be reduced by improving nursing care for osteoporosis (270). First, we should strengthen the publicity and education on osteoporosis and improve people’s awareness of preventative measures (271). Second, this study found that undernutrition and malnutrition were common in senile patients with osteoporosis, especially in patients with hip fractures. With increasing age, calcium intake, intestinal absorption of calcium, intestinal epithelial absorption ability to adapt to low calcium intake, exposure to sunlight, and the skin’s ability to produce vitamin D decrease. Chronic secondary hyperparathyroidism caused by calcium and vitamin D deficiencies leads to increased bone turnover, which, in turn, leads to osteoporosis. Therefore, protein and calcium intake should be strengthened in patients with osteoporosis (272). Some studies have found that, to a certain extent, a lack of proper physical exercise and less sunlight leads to insufficient vitamin D, decreased calcium absorption in the body, and unhealthy psychological conditions, such as anxiety and depression. Therefore, proper sun exposure should be obtained and aerobic exercises should be performed to supply vitamin D to the body (273). In addition, every patient with osteoporosis should increase compliance under the professional guidance of doctors and correct unhealthy living habits and styles, such as sedentary habits, smoking, and paying attention to diet. Measures to prevent falls in the daily life of patients with osteoporotic fractures should be implemented as soon as possible without delaying the best treatment time (274).

## 6 Summary and prospects

The number of patients with RA and PMOP increases rapidly every year, and bone dysfunction caused by RA and PMOP not only exerts puts great pressure on families and society, but also seriously reduces the quality of life of patients (275, 276). Currently, conventional treatments for RA and PMOP can no longer meet people’s needs, and the development of new therapeutic drugs and methods is urgently needed. The emergence of bone immunology and the development of traditional Chinese medicine have provided insights for the treatment of patients with RA and PMOP. Th17 and Treg cells, which differentiate from CD4+T cells, play important roles in bone immune diseases. For example, Th17 cells secrete inflammatory factors in RA, which not only induce inflammatory reactions, but also promote cartilage destruction and OC differentiation directly or indirectly. Moreover, the decrease in oestrogen in PMOP increases the differentiation of CD4+T cells into Th17 cells which shifts the Th17/Treg ratio, Th17 cells and their pro-inflammatory factors induce OC formation, cause bone loss, and lead to an imbalance in bone remodelling. Tregs can induce the differentiation of BMSCs into OB and promote their proliferation and differentiation by secreting related cytokines. They can also inhibit the differentiation of Th17 cells by inhibiting the cytokines secreted by Th17 cells, which inhibit not only inflammatory reactions but also directly and indirectly decrease OC production, and they also play an important role in regulating bone immune diseases, such as RA and PMOP. Therefore, alterations in the balance of Th17/Treg cells is one of the main pathogeneses of bone immune diseases, such as RA and PMOP.

This review analyses the regulatory mechanisms associated with related inflammatory factors, cytokines, transcription factors, and signalling pathways in the inflammatory response and bone remodelling in RA and PMOP based on their interference with Th17 and Treg differentiation. Moreover, Th17 and Treg regulate the balance of Th17/Treg cells by secreting related cytokines. In addition, this review summarises the current routine treatment status of RA and PMOP and focuses on new therapeutic drugs that can target and regulate Th17/Treg imbalance, including probiotic therapy, traditional Chinese medicine compound therapy, traditional Chinese medicine monomer therapy, new targets, new drugs, and new targeted methods. In addition to treatment, this article further reveals the influence of RA and PMOP on patients’ quality of life and identifies patient-centred nursing methods.

In summary, although the effectiveness of regulating Th17/Treg balance in the treatment of immune bone diseases has been confirmed, it is necessary to identify more accurate treatment methods that consider the complexity and uncertainty of the pathogenesis of immune bone diseases. We hope that immunotherapy, which focuses on regulating the Th17/Treg balance, will become a powerful tool against immune bone diseases through continuous research and development.

## Author contributions

XW: Writing – original draft. BS: Writing – original draft. YW: Writing – review & editing, Resources. PG: Writing – review & editing, Resources. JS: Writing – review & editing, Resources. WC: Writing – review & editing, Visualization. ZX: Writing – review & editing, Visualization. YX: Visualization, Writing – review & editing. ZL: Visualization, Writing – review & editing. FA: Conceptualization, Writing – review & editing. CY: Conceptualization, Writing – review & editing.
